# The Transcriptional Cycle Is Suited to Daytime N_2_ Fixation in the Unicellular Cyanobacterium “*Candidatus* Atelocyanobacterium thalassa” (UCYN-A)

**DOI:** 10.1128/mBio.02495-18

**Published:** 2019-01-02

**Authors:** María del Carmen Muñoz-Marín, Irina N. Shilova, Tuo Shi, Hanna Farnelid, Ana María Cabello, Jonathan P. Zehr

**Affiliations:** a Ocean Sciences Department, University of California, Santa Cruz, Santa Cruz, California, USA; b Departamento de Bioquímica y Biología Molecular, Campus de Excelencia Internacional Agroalimentario, Universidad de Córdoba, Córdoba, Spain; c State Key Laboratory of Marine Environmental Science, Xiamen University, Xiamen, China; d Centre for Ecology and Evolution in Microbial Model Systems (EEMiS), Linnaeus University, Kalmar, Sweden; University of Southern California; Pennsylvania State University; University of Warwick

**Keywords:** cyanobacteria, diel cycle, marine microbiology, nitrogen fixation, symbiosis, whole-genome expression

## Abstract

Symbiosis between a marine alga and a N_2_-fixing cyanobacterium (*Cyanobacterium* UCYN-A) is geographically widespread in the oceans and is important in the marine N cycle. UCYN-A is uncultivated and is an unusual unicellular cyanobacterium because it lacks many metabolic functions, including oxygenic photosynthesis and carbon fixation, which are typical in cyanobacteria. It is now presumed to be an obligate symbiont of haptophytes closely related to Braarudosphaera bigelowii. N_2_-fixing cyanobacteria use different strategies to avoid inhibition of N_2_ fixation by the oxygen evolved in photosynthesis. Most unicellular cyanobacteria temporally separate the two incompatible activities by fixing N_2_ only at night, but, surprisingly, UCYN-A appears to fix N_2_ during the day. The goal of this study was to determine how the unicellular UCYN-A strain coordinates N_2_ fixation and general metabolism compared to other marine cyanobacteria. We found that UCYN-A has distinct daily cycles of many genes despite the fact that it lacks two of the three circadian clock genes found in most cyanobacteria. We also found that the transcription patterns in UCYN-A are more similar to those in marine cyanobacteria that are capable of aerobic N_2_ fixation in the light, such as *Trichodesmium* and heterocyst-forming cyanobacteria, than to those in *Crocosphaera* or *Cyanothece* species, which are more closely related to unicellular marine cyanobacteria evolutionarily. Our findings suggest that the symbiotic interaction has resulted in a shift of transcriptional regulation to coordinate UCYN-A metabolism with that of the phototrophic eukaryotic host, thus allowing efficient coupling of N_2_ fixation (by the cyanobacterium) to the energy obtained from photosynthesis (by the eukaryotic unicellular alga) in the light.

## Abstract (precis)

The symbiotic N_2_-fixing cyanobacterium UCYN-A, which is closely related to Braarudosphaera bigelowii, and its eukaryotic algal host have been shown to be globally distributed and important in open-ocean N_2_ fixation. These unique cyanobacteria have reduced metabolic capabilities, even lacking genes for oxygenic photosynthesis and carbon fixation. Cyanobacteria generally use energy from photosynthesis for nitrogen fixation but require mechanisms for avoiding inactivation of the oxygen-sensitive nitrogenase enzyme by ambient oxygen (O_2_) or the O_2_ evolved through photosynthesis. This study showed that symbiosis between the N_2_-fixing cyanobacterium UCYN-A and its eukaryotic algal host has led to adaptation of its daily gene expression pattern in order to enable daytime aerobic N_2_ fixation, which is likely more energetically efficient than fixing N_2_ at night, as found in other unicellular marine cyanobacteria.

## INTRODUCTION

Nitrogen (N_2_)-fixing microorganisms (diazotrophs), which reduce atmospheric N_2_ to biologically available ammonium, are critical components of aquatic and terrestrial ecosystems because they supply fixed inorganic N (1). Cyanobacteria are particularly important in N_2_ fixation because they can fuel the energy-intensive N_2_ reduction reaction using energy supplied by oxygenic photosynthesis. In the oceans, the filamentous, non-heterocyst-forming cyanobacterium *Trichodesmium* and the heterocyst-forming symbiont of diatoms (*Richelia* and related cyanobacteria) were believed to be the major N_2_-fixing microorganisms until the discovery of the unicellular cyanobacteria *Crocosphaera*, *Cyanothece*, and “*Candidatus* Atelocyanobacterium thalassa” (UCYN-A) in the open ocean. *Crocosphaera* and *Cyanothece* are free-living marine cyanobacteria, but UCYN-A is unusual in that it lacks oxygenic photosynthesis and is a symbiont of a haptophyte alga (related to Braarudosphaera bigelowii). UCYN-A symbiosis is geographically widespread and is important in oceanic N_2_ fixation (2 – 5). The UCYN-A genome has been greatly reduced, with massive metabolic streamlining, including the loss of oxygen-evolving photosystem II (PSII), the carbon-fixing enzyme RuBisCO, and the entire tricarboxylic acid (TCA) cycle (6). UCYN-A has been shown to supply fixed N to the haptophyte in exchange for fixed carbon (4, 7), but it is not known how these two single-celled organisms coordinate metabolism and cell growth over the daily division cycle.

N_2_ fixation requires energy and reductant, but the nitrogenase enzyme is inactivated by oxygen (O_2_). Cyanobacteria generally have access to sufficient energy from photosynthesis but require mechanisms for avoiding inactivation of nitrogenase and N_2_ fixation by ambient oxygen (O_2_) or by the O_2_ evolved through photosynthesis. *Trichodesmium* and heterocyst-forming cyanobacteria such as *Richelia* and *Nostoc* fix N_2_ during the day, whereas the free-living unicellular genera *Crocosphaera* and *Cyanothece* fix N_2_ at night. Interestingly, the symbiotic UCYN-A strain appears to fix N_2_ during the day (8 – 10), in contrast to most other unicellular marine N_2_-fixing cyanobacteria, such as *Crocosphaera* and *Cyanothece*.

The processes of N_2_ fixation and photosynthesis in cyanobacteria are regulated daily to increase cellular fitness and ecological competitiveness (11 – 13). Most cyanobacteria have circadian rhythms (11, 14, 15) that are involved in controlling daily cycles of gene transcription and protein synthesis by signal transduction pathways involving the circadian clock *kai* genes. UCYN-A lacks two (*kaiA* and *kaiB*) of the three *kai* genes known in most other cyanobacteria, whereas the non-N_2_-fixing cyanobacterium *Prochlorococcus* lacks only *kaiA*. Thus, the daily whole-genome expression pattern in UCYN-A is of interest to determine if there are daily patterns similar to those in all other cyanobacteria compared to evolutionarily related unicellular cyanobacteria.

We used a whole-genome transcription array that targets two genetically distinct uncultivated sublineages of UCYN-A (UCYN-A1 and UCYN-A2), which have similar but genetically distinct hosts. We compared the UCYN-A whole-genome diel transcription patterns to those of *Cyanothece* sp. ATCC 51142 (16) and Crocosphaera watsonii WH 8501 (17) (both unicellular nighttime N_2_ fixers) and of Trichodesmium erythraeum IMS101 (a filamentous non-heterocyst-forming daytime N_2_ fixer). We also compared their expression levels to whole-genome expression of *Prochlorococcus* sp. MED4 (18) (a marine non-N_2_ fixer) in order to determine how UCYN-A gene expression levels compare to the general cyanobacterial gene expression levels in a sympatric open-ocean species. We found that many genes in UCYN-A have distinct diel expression patterns and that UCYN-A has unusual gene expression patterns in comparison to unicellular N_2_-fixing cyanobacteria that fix N_2_ in the dark; however, it shares some general patterns with daytime N_2_-fixing cyanobacteria, with heterocysts of heterocyst-forming cyanobacteria, and with unicellular non-N_2_-fixing cyanobacteria. Results suggest that optimal metabolism for open-ocean cyanobacteria is aligned to the light period and that symbiosis has enabled the unicellular UCYN-A to shift N_2_ fixation to the daylight period.

## RESULTS AND DISCUSSION

### UCYN-A has a daily rhythm of gene transcription.

UCYN-A has clear diel patterns of gene transcription, with a large fraction of genes that had periodicity of transcript levels over the dark and light periods (27%).

About a third (31%) of the genes in the UCYN-A genome targeted by the array were transcribed at detectable levels (365 of 1,194 total genes in UCYN-A1 and 394 of 1,244 total genes in UCYN-A2) (see Table S1 in the supplemental material). Approximately 85% of these genes had differences in transcript levels between the dark and light periods, accounting for 27% of the total genes in each strain (Tables S1 and S2). C. watsonii, *Cyanothece* sp., and *Trichodesmium* cultures also had a large fraction of genes with changes in transcript levels between the dark and light periods (39% in C. watsonii, 20% in *Cyanothece* sp., and 34% in *Trichodesmium*) (Tables S1 and S2).

10.1128/mBio.02495-18.5 TABLE S1 Genes targeted and detected in the transcriptomic analysis for each organism. The first line shows the total number of genes targeted for the microarray analysis. The second line shows the number of the genes transcribed at detectable levels and the third line the number of diel genes. Percentages of genes determined by comparing the total genes targeted in the microarray in each organism are shown at the bottom. Download Table S1, DOCX file, 0.1 MB. Copyright © 2019 Muñoz-Marin et al. 2019 Muñoz-Marin et al. This content is distributed under the terms of the Creative Commons Attribution 4.0 International license.

10.1128/mBio.02495-18.6 TABLE S2 List of genes with periodic transcriptional patterns for all studied cyanobacteria based on Fourier scores. Genes with a FDR of <0.25 were selected and are represented in this table for further comparison. Download Table S2, XLS file, 1.3 MB. Copyright © 2019 Muñoz-Marin et al. 2019 Muñoz-Marin et al. This content is distributed under the terms of the Creative Commons Attribution 4.0 International license.

The UCYN-A transcription values (log_2_ transformed) ranged from 2 to 13.5, with a median of 6.0. In both sublineages, the genes coding for nitrogenase (*nif*), F_0_F_1_-ATP synthase (*atpA* and *atpB*) and the cytochrome *b*_6_*f* complex (*petB*, *petC*, *petF*, and *petL*) and the photosynthetic gene *psaC* were the most highly transcribed among all of the detected genes (Table S5). The transcript levels of the same genes were also high for both sublineages in metatranscriptomes collected during the TARA expedition in the South Atlantic Ocean (19).

10.1128/mBio.02495-18.9 TABLE S5 Detailed lists of normalized transcription values and description for all genes in all studied cyanobacteria. There are two spreadsheets for each organism: “Data” and “Description.” The “Data” spreadsheet shows the normalized transcription values for each time point and the mean expression. Eight points were selected for UCYN-A (L6, L9, D3, D6, 2D12, 2L3, 2L9, and 2L12), 9 points for T. erythraeum (D12, L3, L6, L9, L12, D3, D6, D9, and 2D12), 6 points for *Cyanothece* sp. ATCC 51142 (L2, L6, L10, D2, D6, and D10), 8 points for C. watsonii WH 8501 (D11, L1, L6, L11, D1, D6, 2D11, and 2L1) and 19 points for *Prochlorococcus* sp. MED4 (D12 to 2L12 [representing time points occurring every 2 h]). “L” and “D” stand for the light period and the dark period, respectively, “2L” and “2D” for the second light-dark cycle, and the number that follows for the hour corresponding to the time of entry into the light or dark period. The “Description” spreadsheet shows the annotation and pathways of each gene for each organism. Download Table S5, XLSX file, 2.6 MB. Copyright © 2019 Muñoz-Marin et al. 2019 Muñoz-Marin et al. This content is distributed under the terms of the Creative Commons Attribution 4.0 International license.

**FIG 1:**
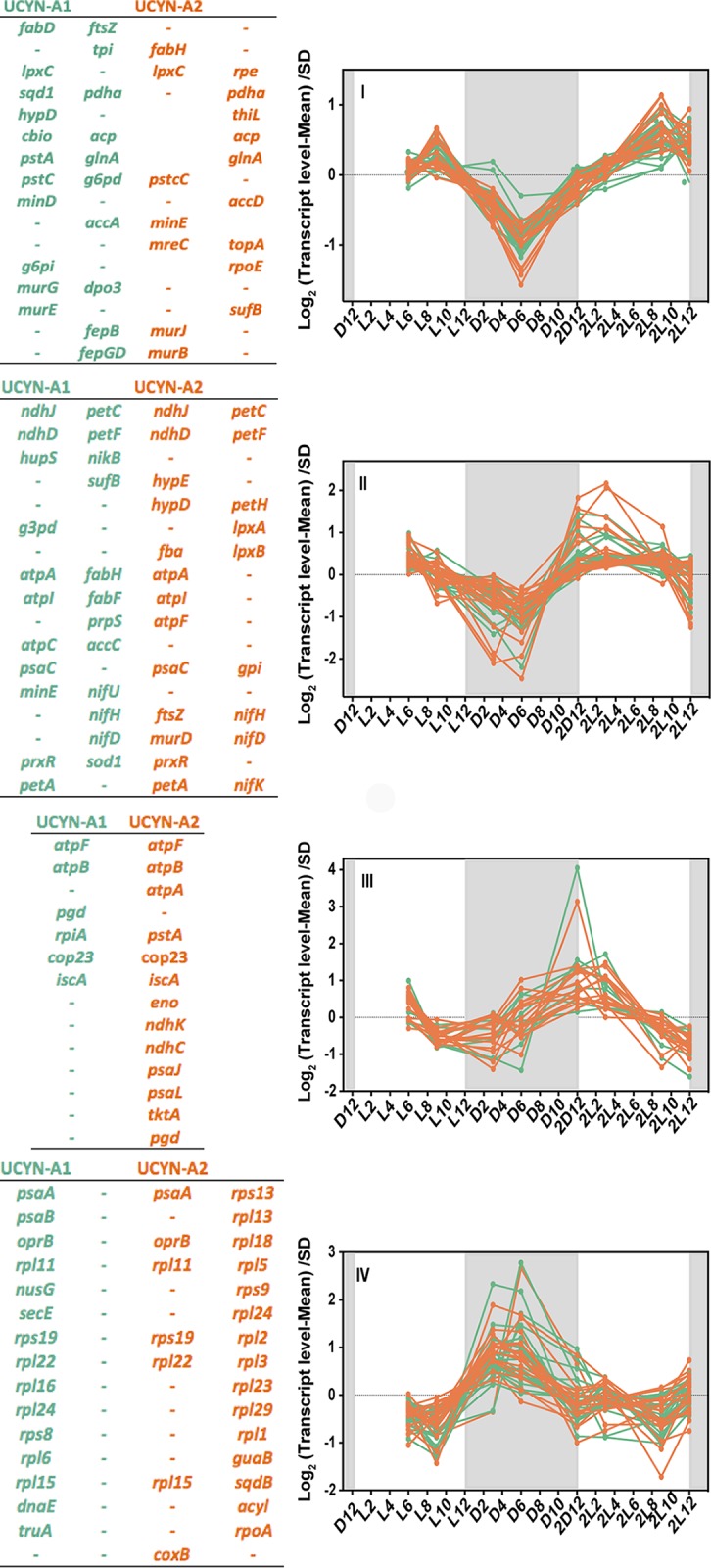
Four different clades (indicated by I-IV) based on Pearson correlation of the transcription profiles of UCYN-A1 and UCYN-A2 genes over light-dark cycles. The transcription value of each gene at each time point was normalized to the mean at all time points and divided by the standard deviation (SD) (*y* axis, log 2 scale). The *x* axis represents time points where “D” and “L” stand for dark and light, respectively, followed by a number corresponding to the hour of entry into the light or dark period. The second light-dark cycle is indicated as “2D” followed by a number corresponding to the hour of entry into the light or dark period. The shaded area represents the dark period. In each cluster, the most representative genes are listed in the table attached to the plot. UCYN-A1 genes are coded in green and UCYN-A2 genes are coded in orange.

The two UCYN-A sublineages were similar with respect to the periodicity of their transcript levels, despite divergence in gene sequences at the amino acid level (genome-wide average of 14%), in cell morphology (19), and in genome size (Fig. 1). There were four gene clusters as determined based on the time of day exhibiting the highest relative transcript level (Fig. 1). Cluster I had the highest relative transcript level during the day (with the maximum seen 10 h into the light period) and included genes involved in cell division (e.g., *ftsZ*, *murG*, *minE*, and *murB*), DNA replication (e.g., *topA*, *rpoE*, and *DPO3B*), ABC transportation (e.g., *nikA*, *nikB*, *pstC*, and *cbiO*), and carbohydrate and lipid metabolism (e.g., *pdhA*, *pgi*, *fabG*, and *fabH*) and a few photosynthesis genes (*petL*, *psaD*, and *ccsB*). The transcription levels for the *petL* gene, encoding subunit 6 of the cytochrome *b*_6_*f* complex, and for the only nitrogen fixation-related gene in this cluster (*nifK*) showed a substantial (more than 3-fold) change at that time.

The transcript abundances of genes from clusters II and III had similar patterns, with an increase before sunrise and a decrease during the dark period. The highest relative transcript levels for the genes in clusters II and III were seen at 4 h and 1 h after sunrise, respectively, and included genes involved in nitrogen fixation (*nifHDK* operon) that increased 4-fold in transcription during the light period. However, these clusters also included genes involved in oxidative phosphorylation (e.g., NADH dehydrogenase subunit-related genes and ATP synthase-related genes) and in carbohydrate catabolism such as those involved in glycolysis (e.g., *gap1*, *fbaA*, *pgi*, and *eno*), in the pentose phosphate pathway (*opcA* and *zwf*), and in photosynthesis (e.g., cytochrome *b*_6_*f* complex subunit genes). In most cyanobacteria, genes encoding proteins involved in carbohydrate catabolism are highly transcribed during the night and are essential for survival under dark conditions.

The gene with the most dramatic difference in transcript levels between the light and dark periods encoded membrane protein COP23 (23-kDa circadian oscillating protein), which had a change in transcript abundance of more than 5-fold in both UCYN-A strains (Fig. 1). COP23, a protein which may have a critical role in membrane function, has been detected only in nitrogen-fixing cyanobacteria (20).

Cluster IV had genes with the highest transcript level during the night and the lowest during the day and included genes encoding photosystem I (PSI) subunits and a carbohydrate porin (*oprB*) and also genes encoding ribosomal proteins with 2- and 4-fold changes during the night period. Cluster IV had the lowest number of genes compared with the other clusters. Surprisingly, the PSI genes (*psaA* and *psaB*) were expressed during the night, as seen in many anoxygenic phototrophic bacteria (21), whereas these genes are expressed during the day in most oxygenic cyanobacteria (including mats) (22).

The results show that UCYN-A has a daily rhythm of gene expression with strong periodicities of transcript levels over the diel cycle. Daily patterns of gene transcription in cyanobacteria are typically regulated by a circadian rhythm mediated by *kai* gene products (11). Rhythmic daily transcription patterns are still possible without the full suite of *kai* genes; for example, the marine cyanobacterium *Prochlorococcus* sp. MED4 lacks one of the circadian genes, *kaiA*, and yet it maintains strong diel gene transcription patterns (18). However, *Prochlorococcus* sp. PCC 9511 loses the typical periodicities of the circadian clock under conditions of continuous light (23). In the case of UCYN-A, it lacks two of the three *kai* genes (24), which is unique among cyanobacteria; furthermore, the *kaiC* gene was not transcribed at detectable levels. It is unclear what controls the UCYN-A diel gene expression pattern, but it could be that (i) there are unidentified components of a clock and signal transduction pathway or (ii) the pattern could be driven by the physiological differences between light and dark conditions and might primarily be driven by energy supplied by the eukaryotic partner. It is possible that the diel transcription patterns in UCYN-A are primarily regulated by the daily host metabolism, which itself is likely to be circadian. However, it is not yet known whether the UCYN-A diel cycle is maintained under constant conditions in UCYN-A or whether the diel pattern is maintained in the absence of the partner alga.

### UCYN-A transcription patterns are similar to those of aerobic marine daytime N_2_-fixers and non-N_2_-fixers.

**FIG 2:**
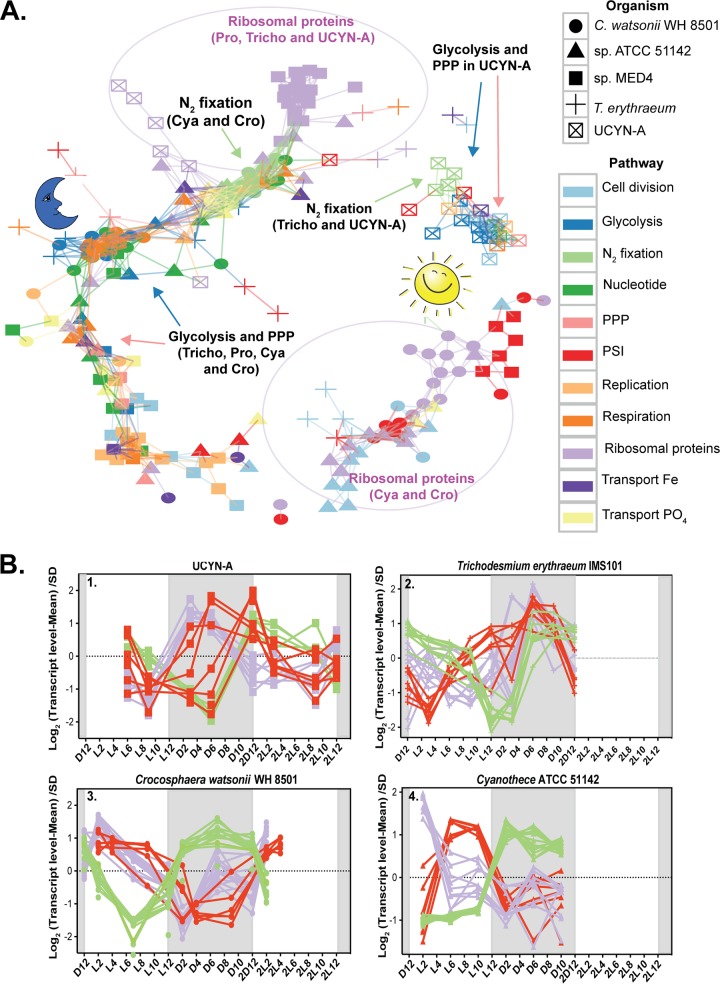
(A) Transcriptional network based on Pearson correlation of gene transcription over the diel cycle in all studied cyanobacteria. The genes are connected if correlation coefficient for their transcription patterns is higher than 0.5. The genes shown are diel genes with variable transcription patterns among the studied cyanobacteria. The arrows point to genes for glycolysis, the pentose phosphate pathway, and N_2_ fixation in the studied diazotrophs. The purple circles demarcate genes for ribosomal proteins included in the analysis. Abbreviations: *Prochlorococcus* sp. MED4 (Pro), *Cyanothece* sp. ATCC 51142 (Cya), C. watsonii WH 8501 (Cro), T. erythraeum (Tricho), pentose phosphate pathway (PPP), photosystem I (PSI). (B) Four time course plots for the N_2_-fixing cyanobacteria, showing the diel transcription patterns of photosystem I genes, N_2_ fixation genes, and genes for ribosomal proteins.

UCYN-A had diel whole-genome expression patterns that were different from those of phylogenetically closely related unicellular cyanobacteria (17). Only a few genes (such as those encoding ATP synthase) had the same daily pattern among all cyanobacteria, presumably differing because of physiology (e.g., N_2_-fixing or not). The unicellular cyanobacteria C. watsonii WH 8501 and *Cyanothece* sp. ATCC 51142, which fix N_2_ during the night, expressed many genes in a pattern opposite that seen with daytime N_2_-fixing T. erythraeum and UCYN-A (Fig. 2; see also Tables S3, S4, and S5). Interestingly, the diel transcription patterns of N_2_ fixation and PSI genes in UCYN-A were opposite those in *Cyanothece* sp. ATCC 51142 and C. watsonii WH 8501 and similar to those in T. erythraeum (Fig. 2; see also Tables S3, S4, and S5).

10.1128/mBio.02495-18.7 TABLE S3 Gene orthologs among all studied cyanobacteria. The orthologs were identified as the best hits in reciprocal blastn searches for each pair of genomes. Orthologs of UCYN-A diel genes detected in the other cyanobacteria are listed in sheets 2 to 5. The percentages of diel genes shared between UCYN-A and the respective cyanobacterial species were calculated relative to the total gene orthologs identified by blastn for the two cyanobacterial species. Download Table S3, XLSX file, 0.4 MB. Copyright © 2019 Muñoz-Marin et al. 2019 Muñoz-Marin et al. This content is distributed under the terms of the Creative Commons Attribution 4.0 International license.

10.1128/mBio.02495-18.8 TABLE S4 Genes selected for network analysis. A minimum of 50% of genes of UCYN-A with diel transcription patterns is represented in the figure. The same genes or genes in the same pathway were represented for the rest of cyanobacteria. The last column (“Genes selected for network”) indicates the genes that were used to build the network analysis whose results are presented in Fig. 2 and 6. Download Table S4, XLS file, 1.2 MB. Copyright © 2019 Muñoz-Marin et al. 2019 Muñoz-Marin et al. This content is distributed under the terms of the Creative Commons Attribution 4.0 International license.

As observed for the activity of nitrogenase, it has been demonstrated that levels of *nif* transcripts and the biosynthesis of different components of the nitrogenase complex are very sensitive to O_2_ (22, 25 – 27), most likely as a consequence of the need to avoid energy losses associated with the degradation of this enzyme by O_2_. Thus, the different patterns observed in the genes involved in N_2_ fixation in the cyanobacteria studied here presumably are due to the different mechanisms used to protect the nitrogenase complex from the O_2_ produced by photosynthesis. T. erythraeum and UCYN-A showed maximum transcript levels of the nitrogenase and PSI genes just prior to dawn but maintained high levels of transcripts for both sets of genes during the day. The peak of transcript levels just before dawn was likely due to the advantage of synthesizing nitrogenase in preparation for N_2_ fixation in the early hours of the day (28).

The diel expression patterns of genes that are unrelated to N_2_ fixation in the aerobic daytime N_2_ fixers (T. erythraeum and UCYN-A) were also more similar to those of non-N_2_-fixing sympatric cyanobacteria of the genus *Prochlorococcus* and of heterocysts of heterocyst-forming cyanobacteria than to those of the nighttime N_2_-fixing cyanobacteria (C. watsonii and *Cyanothece* sp.). The transcript levels of genes encoding ribosomal proteins in both UCYN-A and T. erythraeum were higher during the night, probably because the reduced nitrogen required for the synthesis of new proteins was obtained during the day (Fig. 2; see also Tables S4 and S5). Similar patterns were observed in *Prochlorococcus*, with higher transcript levels seen during the night (Fig. 2; see also Tables S4 and S5), while genes encoding ribosomal proteins in C. watsonii WH 8501 and *Cyanothece* sp. ATCC 41142 had maximum transcript levels during the day (Fig. 2; see also Tables S4 and S5). Intriguingly, these results imply that both UCYN-A and T. erythraeum have adopted daytime gene transcription patterns for the main metabolic pathways, minimizing cellular processes in the dark. The nighttime patterns of the transcript levels of the ribosomal proteins (genes) would make it possible to have proteins synthesized in order to make the most efficient use of the light period, as seen in *Prochlorococcus*. Because UCYN-A and *Trichodesmium* are likely the two most abundant N_2_-fixing cyanobacteria in the open ocean, it appears that direct coupling of N_2_ fixation to photosynthesis is important in the oligotrophic environment (as long as low oxygen concentrations are maintained in the cell).

Phosphorus is a vital element for cellular energetics and growth and is acquired by oceanic bacterioplankton primarily as phosphate (29 – 31). The UCYN-A phosphate ABC transporter had the same diel pattern as that in *Trichodesmium* for genes involved in DNA replication, with higher transcript levels during the day (Table S5) but with maximum transcript abundances during the late afternoon in *Crocosphaera* and *Cyanothece* (17, 32). The presence of high levels of phosphate transporters during the day could meet the increased demand for inorganic phosphate (33, 34) during DNA replication, which occurs during the day in UCYN-A and *Trichodesmium*. Similar patterns were observed in the heterocyst-forming *Richelia* species, with peak expression of P acquisition genes at approximately 15:00 h, suggesting that the apparent rhythmicity of P acquisition could be a common feature of daytime N_2_ fixers (35).

**FIG 3:**
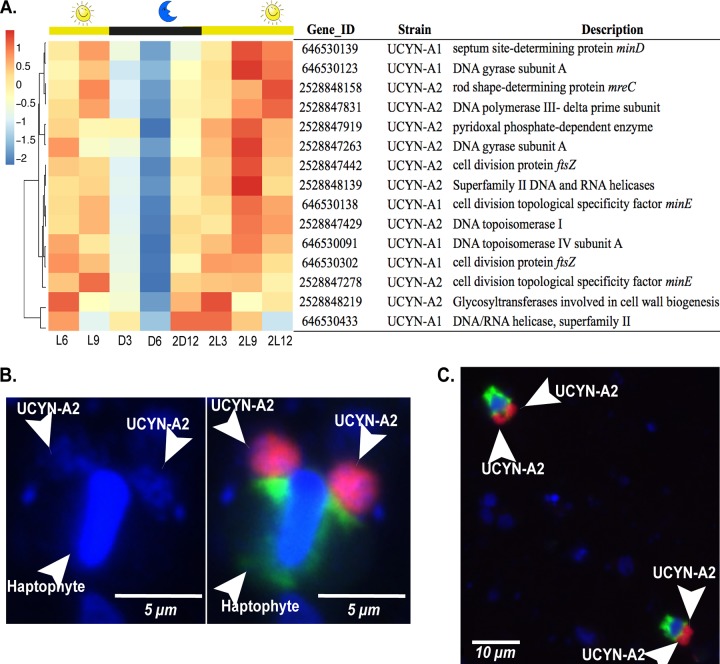
Transcription of genes for replication and cell division in UCYN-A. (A) (Upper panel) Diel transcription patterns for cell division and replication genes in UCYN-A1 and UCYN-A2 over the light-dark cycle. Hierarchical clustering of genes was based on Pearson correlations between their transcription profiles. The transcription values of the genes were standardized at each time point, and the blue-red scale shows by how many standard deviations a transcription value was lower (blue) or higher (red) than the mean transcription values over the diel cycle (Z score). The Gene ID and the gene product corresponding to each gene for UCYN-A1 and UCYN-A2 are shown. Time periods are indicated on the *x* axis as “L” (light) and “D” (dark) followed by a number corresponding to the hour after the sunrise and sunset periods started. The second light-dark cycle is indicated as “2D” followed by a number corresponding to the hour of entry into the light or dark period. (Lower panel) Epifluorescence micrographs of dividing UCYN-A2 detected with CARD-FISH (19). (B) Two big clusters of UCYN-A2 cells and the attached haptophyte host. (Left panel) The nucleus of the host and the UCYN-A2 cells were visualized with DAPI stain (blue). (Right panel) The UCYN-A2 strain (red) and its haptophyte host (green). (C) Two different associations of UCYN-A2 with its haptophyte dividing in samples from Scripps Pier.

The factor initiating DNA replication, DnaA, is a protein that is highly conserved in prokaryotes although it is absent in red algae, the cyanobacterial symbiont *Nostoc azollae* (36), and also the spheroid bodies of diatoms (37). The genome of UCYN-A lacks the *dnaA* gene as well. Recent studies suggested that DnaA is not essential for DNA replication and that the lack of *dnaA* could suggest a preadaptation of the genome to enable the symbiosis (38). In UCYN-A and T. erythraeum, genes for DNA replication (*dnaE* and the RNase H_1_ gene), DNA topoisomerases, DNA gyrases, and cell division (*ftsZ*, *mre*, and *min*) showed maximum transcript levels during the day (i.e., after midday) and minimum levels at night (Fig. 3A; see also Fig. S1 in the supplemental material). In contrast, the nighttime N_2_-fixing *Cyanothece* sp. ATCC 51142 and C. watsonii WH 8501 confined cell division to the period of transition from dark to light at sunrise. The temporal delay in cell division in *Cyanothece* and *Crocosphaera* has been suggested to reflect the need to recover energy reserves with light-derived energy after nighttime metabolic activity (39). The similarity of the pattern in UCYN-A to that in *Trichodesmium* is consistent with UCYN-A shifting metabolism to the daytime.

10.1128/mBio.02495-18.2 FIG S1 Transcription patterns in *T. erythraeum* for the cell division and replication genes over the light-dark cycle. Hierarchical clustering of genes was based on Pearson correlation of gene transcription profiles. The transcription values fof each gene at each time point were standardized, and the blue-red scale shows by how many standard deviations a transcription value is lower (blue) or higher (red) than the mean transcription values over the diel cycle (Z score). “L” and “D” stand for light and dark, respectively; those notations are followed by the hour corresponding to the time after the light and dark periods started. The second light-dark cycle is indicated as “2D,” and that notation is followed by the number of the hours corresponding to entry into the light or dark period. Download FIG S1, PDF file, 0.8 MB. Copyright © 2019 Muñoz-Marin et al. 2019 Muñoz-Marin et al. This content is distributed under the terms of the Creative Commons Attribution 4.0 International license.

Microscopy counts of B. bigelowii–UCYN-A2 symbiosis were performed eight times during two diel cycles in order to observe the timing of cell division (Fig. 3B and C; see also Table S6). In both diel cycles, single host cells with two associated UCYN-A2 cells (or groups of cells), corresponding to approximately 60% of the total cell counts, were present at night between 21:00 and 03:00 h. The delay observed between the time of the higher transcription levels after midday and the time of actual cell division at h 21:00 may be explained by the need of the cell to coordinate the assembly of the cell division machinery prior to cell division.

10.1128/mBio.02495-18.10 TABLE S6 Abundance of the haptophyte UCYN-A2 association in CARD-FISH samples during two diel cycles at the Scripps Institution of Oceanography, indicating the number of UCYN-A2 clusters per host cell. Download Table S6, DOCX file, 0.2 MB. Copyright © 2019 Muñoz-Marin et al. 2019 Muñoz-Marin et al. This content is distributed under the terms of the Creative Commons Attribution 4.0 International license.

### Unique UCYN-A transcription patterns.

**FIG 4:**
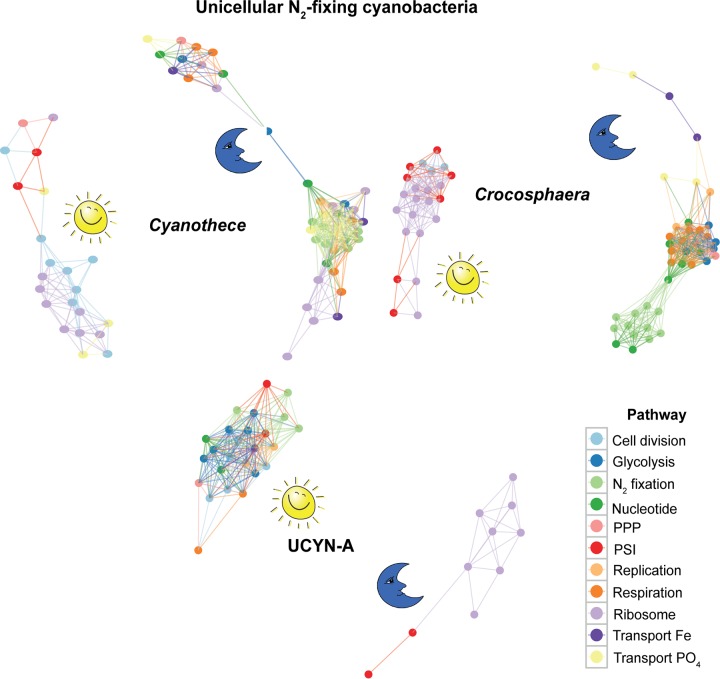
Network showing the Pearson correlation for gene transcriptions in the unicellular N_2_-fixing cyanobacteria *Cyanothece* sp. ATCC 51142 (*Cyanothece*), C. watsonii WH 8501 (*Crocosphaera*), and UCYN-A. Shown here are key genes in major metabolic pathways with distinct diel transcription patterns. The genes are shown as connected if their correlation coefficient for transcription patterns is higher than 0.2. PPP, pentose phosphate pathway; PSI, photosystem I.

**FIG 5:**
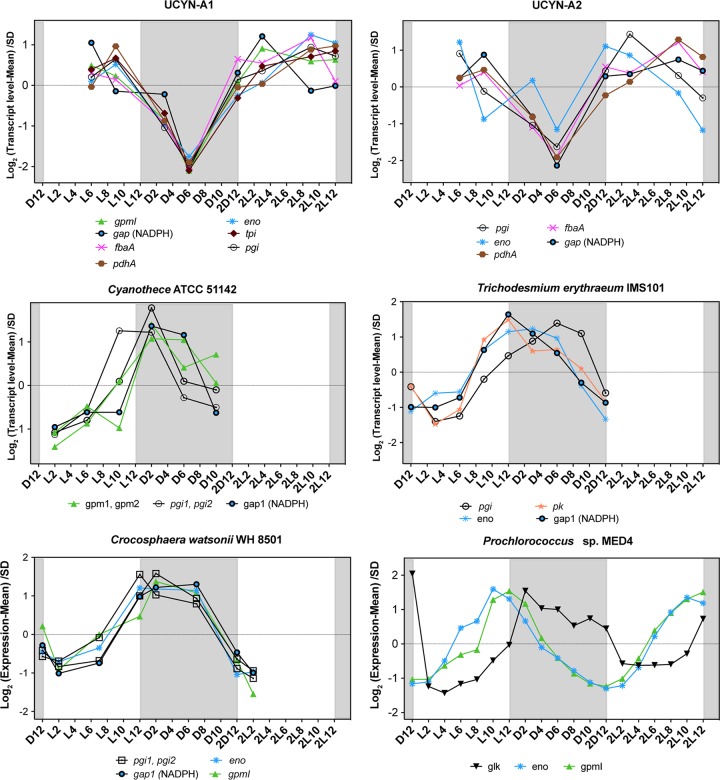
Transcriptional profiles of the genes for glycolysis over light-dark cycles in the cyanobacteria studied here. The transcription value of each gene at each time point was normalized to the mean at all time points and divided by the standard deviation (SD) (*y* axis, log scale). The *x* axis represents time points where “D” and “L” stand for dark and light, respectively, followed by a number corresponding to the hour of entry into the light or dark period. The second light-dark cycle is indicated as “2D” followed by a number corresponding to the hour of entry into the light or dark period. The shaded area represents the dark period.

**FIG 6:**
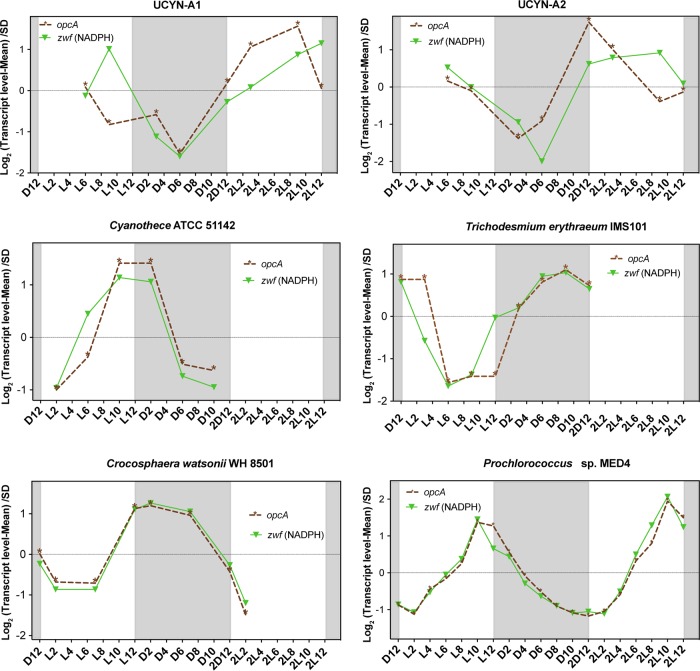
Transcriptional profiles of *opcA* (allosteric effector) and *zwf* (glucose-6-P dehydrogenase) over light-dark cycles in the cyanobacteria studied here. The transcription value of each gene at each time point was normalized to the mean at all time points and divided by the standard deviation (SD) (*y* axis, log scale). The *x* axis represents time points where “D” and “L” stand for dark and light, respectively, followed by a number corresponding to the hour of entry into the light or dark period. The second light-dark cycle is indicated as “2D” followed by a number corresponding to the hour of entry into the light or dark period. The shaded area represents the dark period.

Although many gene transcription patterns in UCYN-A are more similar to those in *Trichodesmium* than to those in other unicellular N_2_-fixing cyanobacteria, some of the patterns were unique to UCYN-A. Such unique gene transcription patterns in the UCYN-A symbiosis may provide clues to possible roles of specific genes involved in adaptation to N_2_-fixing symbiosis, revealing metabolic interdependence between host and symbiont. In order to compare the transcriptomic patterns of these specific genes with those of the rest of the N_2_-fixers, we performed network analysis of the genes using Pearson correlation. Whereas most of the key genes of the major pathways in UCYN-A had higher transcript levels during the day, those of the other unicellular N_2_-fixing cyanobacteria had maximum transcript levels at night (Fig. 4). For example, glycolysis genes in UCYN-A had the highest levels of transcripts at sunrise and midday (maximum light conditions) compared to the other cyanobacteria (Fig. 4 and 5). The metabolic pathway that generates reductant for biosynthesis activities (NADPH), i.e., the pentose phosphate pathway (PPP), had similar patterns. The allosteric effector *opcA*, which redirects carbon flow to the first enzyme of the PPP (glucose-6-P dehydrogenase [*zwf*]) (18, 40), had a periodic transcript level pattern in UCYN-A (Fig. 4 and 6) that was different from that previously reported in other cyanobacteria (41, 42).

**FIG 7:**
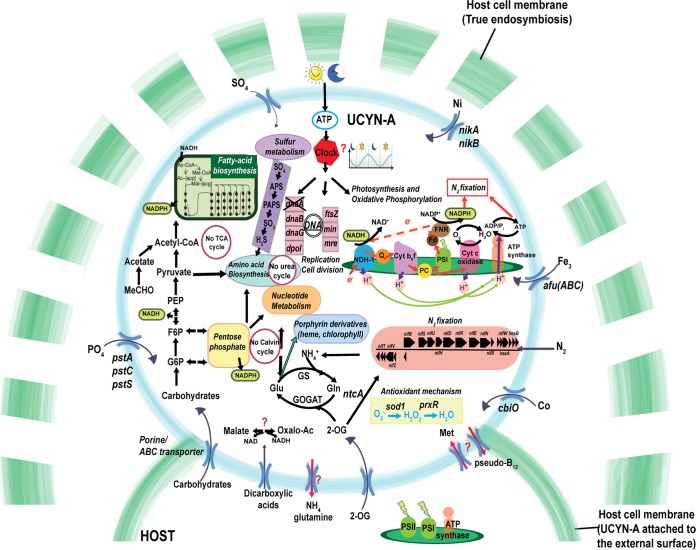
Schematic model of UCYN-A showing the possible main cellular functions, metabolic pathways, and transporters.

N_2_ fixation in UCYN-A depends on the light period for the supply of photosynthate from the host during the day, as well as possibly producing ATP by cyclic photophosphorylation with PSI. Because UCYN-A cannot fix carbon dioxide, it has to obtain reduced carbon compounds in the same way. On the basis of the genome and transcriptomic profiles, we propose a pathway of carbon metabolism for the regeneration of reductant and ATP in UCYN-A that is needed for N_2_ fixation (Fig. 7). Carbohydrate porins or ABC transporters could transport the carbohydrates from the host to the cyanobacteria during the day and the carbon compounds metabolized through the oxidative pentose phosphate (OPP) pathway or glycolysis pathway. Pyruvate is required for generation of reductant for nitrogenase and also to generate acetyl-coenzyme A (acetyl-CoA) for synthesis of fatty acids.

Because UCYN-A lacks photosystem II, which normally supplies electrons to photosystem I by splitting water, UCYN-A needs alternative electron donors if it uses PSI to make the reductant NADPH. The NADH generated by the OPP pathway or by glycolysis could reduce the plastoquinone (PQ) pool via the NDH-1 complex and transfer electrons to ferredoxin though the PQ pool, cytochrome *b*_6_*f* plastocyanin, and the action of PSI. Ferredoxin could deliver electrons to the ferredoxin:NADPH oxidoreductase (FNR), which might supply reductant and ATP directly to the dinitrogenase reductase. To increase the ATP/e^−^ ratio, UCYN-A can redirect electrons from PSI to NDH-1 in cyclic phosphorylation. This mechanism to supply nitrogenase with electrons was proposed years ago for heterocysts (43).

Together, the results are consistent with the assumption that UCYN-A uses host-supplied carbohydrates during the day whereas other unicellular cyanobacteria synthesize their own carbohydrates during the day and use them during the evening or at night. The unique distribution of these metabolic processes suggests that UCYN-A has developed the ability to perform light-driven, daytime N_2_ fixation under oxic conditions as a result of symbiosis.

Apart from fixed carbon, several other compounds may be made available to UCYN-A, which may be endosymbiotic and relies on the host for all of its essential nutrients. Interestingly, UCYN-A has the whole pathway for the synthesis of the cyanobacterial type of vitamin B_12_, pseudocobalamin, that can be required for the activity of several vital enzymes in central metabolism (44) (Table S8). Transcription of genes involved in B_12_ synthesis were detected in all cyanobacteria, and some of them had diel patterns (Table S2 and S8). It is unknown if UCYN-A has enzymes that require pseudocobalamin or whether it can be used by the host. However, in order for the host to use pseudocobalamin, it would have to be remodeled in order for it to be accessible to the haptophyte (45). The role of pseudo-B_12_ biosynthesis in UCYN-A is unclear, but the fact that UCYN-A retains this entire pathway, in such a reduced genome, indicates that it is likely to have an important role, perhaps in symbiosis.

10.1128/mBio.02495-18.12 TABLE S8 Detailed list of genes involved in B_12_ metabolism in all studied cyanobacteria. Download Table S8, XLSX file, 0.04 MB. Copyright © 2019 Muñoz-Marin et al. 2019 Muñoz-Marin et al. This content is distributed under the terms of the Creative Commons Attribution 4.0 International license.

It is still unclear how N_2_ fixation in UCYN-A avoids the oxygen evolved by the photosynthetic host alga. There are only two possible pathways for consuming O_2_ in UCYN-A, including aerobic (cytochrome-dependent) respiration and the photocatalyzed reduction of O_2_ to H_2_O in PSI such as occurs in the heterocysts of cyanobacteria like *Nostoc* sp. PCC 7120 (46 – 48). The latter, called the Mehler reaction, results in the production of the superoxide radical O_2_^–^, which is subsequently reduced to water (46, 49).

In UCYN-A, the cytochrome *c* oxidase *coxA* gene was transcribed during the night (cluster IV) but also (rarely) during the day, along with a few N_2_ fixation genes (cluster I) (Fig. 1). Moreover, we also found higher transcript levels during the day for the antioxidant enzyme superoxide dismutase (*sod1*) and two peroxiredoxins (*prxR*), which have the ability to detoxify peroxide (Fig. 1 and 7). Both antioxidants would protect the nitrogenase against the reactive oxygen species produced by UCYN-A or the haptophyte host (Fig. 7).

It is not currently possible to directly determine the oxygen protection mechanisms in this uncultured microorganism because (i) transcription cannot definitively be related to function and (ii) it is not possible to perform physiological experiments with this low-abundance microorganism, which has yet to be obtained in an axenic culture. Consequently, the issue of protection from O_2_ cannot be directly addressed experimentally, but our results suggest that some of the proteins in UCYN-A could help to protect nitrogenase from the O_2_ generated by host photosynthesis.

Because UCYN-A shows genome reduction normally associated with endosymbiosis (e.g., in Paulinella chromatophora [50]), the unique gene transcription patterns of UCYN-A may provide insights into the evolution of endosymbiosis and organellar evolution. Future studies are needed to determine if the rhythm of these patterns is maintained under constant conditions as in the case of a circadian rhythm, whether the host has a circadian rhythm, and/or whether the daily cycle in UCYN-A simply responds to metabolite availability from the host. It will also be interesting to determine how PSI is involved in supporting the energy or reductant requirements of N_2_ fixation. Such experiments will have to await the establishment of a pure culture.

## MATERIALS AND METHODS

### Diel sampling of UCYN-A.

Surface seawater samples for UCYN-A transcription and catalyzed reported deposition-fluorescence *in situ* hybridization (CARD-FISH) analyses were collected using a bucket from the end of the Scripps Institution of Oceanography (SIO) Ellen Browning Scripps Memorial Pier in La Jolla, CA, USA. Two replicates were collected from the bucket at each time point within 48 h between 28 July and 1 August 2014 for transcriptomic analysis and between 3 and 8 May 2016 for CARD-FISH analysis. A total of 16 samples were collected every 3 to 6 h (with two replicates taken at each of eight time points) as follows: 12:00-L6, 15:00-L9, 21:00-D3, 00:00-D6, 06:00-2D12, 09:00-2L3, 15:00-2L9, and 18:00-2L12. (“L” and “D” stand for the light period and the dark period, respectively, “2L” and “2D” for the second set of light-dark cycles, and the final number for the hour corresponding to entry into the light or dark period.)

For the CARD-FISH assay, 190 ml of seawater from each seawater replicate was fixed with 10 ml 37% formaldehyde (final concentration, 1.87% [vol/vol]) at 4°C in the dark for 1 h. After fixation, 100 ml was filtered at a maximum vacuum pressure of 100 mm Hg onto a 0.6-μm-pore-size, 25-mm-diameter polycarbonate membrane filter (Millipore Isopore; EMD Millipore, Billerica, MA, USA) with a support filter consisting of a 0.8-μm-pore-size, 25-mm-diameter polycarbonate cellulose acetate membrane (Sterlitech Corporation, Kent, WA, USA). The filters were kept at −80°C until processing.

Samples for RNA extraction were collected by filtering a total of 500 ml from each seawater replicate through 0.22-μm-pore-size, 47-mm-diameter Supor filters (Pall Corporation, Port Washington, NY, USA) using a peristaltic pump. Filters were placed in sterile 2-ml bead-beating tubes with sterile glass beads, flash-frozen in liquid nitrogen, and stored at −80°C until extraction.

### Double CARD-FISH assay.

The double CARD-FISH assay was carried out following the protocol designed by Cabello et al. (3) and Cornejo-Castillo et al. (19). Details for all of the probes, competitors, and helpers used in this work are compiled in Table S7 in the supplemental material. More details are provided in Text S1 in the supplemental material. Microscopic evaluation and counting was performed with a Carl Zeiss Axioplan-2 imaging fluorescent microscope (Zeiss, Berlin, Germany) in 3 transects (8.0 by 0.1 mm^2^ each) across the filter piece. Cell dimensions were estimated using AxioVision 4.8 and Image J software (51).

10.1128/mBio.02495-18.1 TEXT S1 Extended description of materials and methods described in the main text. Download Text S1, DOCX file, 0.06 MB. Copyright © 2019 Muñoz-Marin et al. 2019 Muñoz-Marin et al. This content is distributed under the terms of the Creative Commons Attribution 4.0 International license.

10.1128/mBio.02495-18.11 TABLE S7 List of probes utilized in the visualization of UCYN-A associations by double CARD-FISH. Bold letters represent the mismatches between the sequences designed to distinguish UCYN-A1 and UCYN-A2 and their hosts with high specificity. Download Table S7, DOCX file, 0.07 MB. Copyright © 2019 Muñoz-Marin et al. 2019 Muñoz-Marin et al. This content is distributed under the terms of the Creative Commons Attribution 4.0 International license.

### Diel sampling of Trichodesmium erythraeum IMS101 cultures.

Biological triplicate cultures of T. erythraeum were grown in rectangular canted neck polycarbonate cell culture flasks with a 0.2-μm-pore-size vent cap and 225-cm^2^ surface area (Corning Inc., Corning, NY, USA). The cultures were maintained at 26°C on a 12h/12h light/dark cycle at 50 μmol quanta m^−2^ s^−1^ in YBCII media (52) supplemented with 2.8 μmol liter^−1^ ferric ammonium citrate. The light was turned on at h 7:00 and off at h 19:00. The cultures were diluted 10-fold from the inoculum and were verified to be axenic by staining with DAPI (4′,6-diamidino-2-phenylindole) and visualizing cells under an epifluorescence microscope (Carl Zeiss, Thornwood, NY, USA). Growth and cell density were monitored until the cultures reached the exponential phase (∼10 to 14 days after inoculation), during which the cells were harvested for the diel transcription assay. Samples were taken at 3-h intervals starting at the onset of the light period and continuing until the end of the dark period for a total of 24 h. A total of 27 samples were collected from the following nine time points: 7:00-D12, 10:00-L3, 13:00-L6, 16:00-L9, 19:00-L12, 22:00-D3, 1:00-D6, 4:00-D9, and 7:00-2D12 (where “L” and “D” stand for the light period and the dark period, respectively, “2D” for the second light-dark cycle, and the final number for the hour corresponding to entry into the light or dark period). At each time point, 200 ml of each of triplicate cultures (replicates from different flasks) was filtered using a 5-μm-pore-size, 47-mm-diameter polycarbonate membrane filter (Osmonics, Minnetonka, MN, USA). The filters were immediately frozen in liquid nitrogen and stored at −80°C until processing.

### RNA extraction and processing for hybridization to the microarray.

Environmental RNA containing transcripts from UCYN-A cells was extracted using an Ambion RiboPure Bacteria kit (Ambion, Thermo Fisher), with modifications that included mechanical lysis using glass beads (Biospec, Bartlesville, OK). The extracted RNA was treated with a Turbo-DNA-free DNase kit (Ambion, Thermo Fisher) to remove genomic DNA. Sufficient environmental RNA was obtained for two replicates at 4 sampling times (L6, L9, D3, and 2L12) as follows: L6-1, L6-2, L9-1, L9-2, D3-1, D3-2, 2L12-1, and 2L12-2.

Total RNA for T. erythraeum was extracted using an Ambion RiboPure Bacteria kit (Ambion, Thermo Fisher), followed by in-solution DNase digestion with an RNase-free DNase kit and on-column cleanup with an RNeasy MiniElute kit (Qiagen, Valencia, CA, USA).

RNA purity, concentration, and quality were determined using a NanoDrop 1000 instrument (Thermo Scientific, Waltham, MA, USA), a 2100 Bioanalyzer (Agilent Technologies, Santa Clara, CA, USA), and an RNA 6000 Nano kit (Agilent Technologies). Only samples with RNA integrity values of >7.0 and ratios of *A*_260_/*A*_230_ and *A*_260_/*A*_280_ of ≥1.8 were processed further.

Double-stranded cDNA (ds-cDNA) was synthesized from environmental RNA samples that contained UCYN-A and amplified following the procedure previously described by Shilova et al. (53). Briefly, 400 ng RNA from each sample was used, and 1 μl of a 1:100 dilution (corresponding to 4.7 aM of ERCC-0016) of RNA spike-in mix 1 (External RNA Control Consortium [54]) (Ambion) was added before amplification was performed to monitor the technical performance of the assay (54).

Double-stranded cDNA was synthesized and amplified using a TransPlex whole-transcriptome amplification kit (WTA-2; Sigma-Aldrich, St. Louis, MO, USA) and antibody-inactivated hot-start *Taq* DNA polymerase (Sigma-Aldrich). The amplified cDNA was purified with a GenElute PCR cleanup kit (Sigma-Aldrich), and the quality and quantity of ds-cDNA were determined using a NanoDrop 1000 instrument, a 2100 Bioanalyzer, and an Agilent DNA 7500 kit (Agilent Technologies). Total RNA volumes of 400 ng yielded on average 12 μg of ds-cDNA. The labeling and hybridization of cDNA samples (1.0 μg of ds-cDNA) to the microarray were done at the Roy J. Carver Center for Genomics (CCG) Facility (University of Iowa, Iowa City, IA, USA) according to the Agilent Technology protocol for arrays.

For T. erythraeum, at least 30 μg of unamplified total RNA with a concentration of 1.0 μg μl^−1^ per sample was provided for 27 samples. A control sample was generated by mixing equal amounts of total RNA, based on NanoDrop-measured concentrations, from each of the 27 samples, resulting in 28 samples in total. Reverse transcription of the total RNA, labeling of cDNA, and hybridization to the array were performed at the Roche NimbleGen facility according to the protocol of the manufacturer (Roche NimbleGen, Inc., Madison, WI, USA).

### Design of the UCYN-A array.

The UCYN-A oligonucleotide expression array was designed using UCYN-A1 and UCYN-A2 genes and the eArray Web-based tool (Agilent Technology Inc.; https://earray.chem.agilent.com/earray/) similarly to the array design previously described by Shilova et al. (53). The gene sequences were obtained from the National Center of Biotechnology Information (NCBI; https://www.ncbi.nlm.nih.gov). Briefly, six probes of 60 nucleotides (nt) in length were designed for each gene, and a total of 6,618 probes (1,199 genes) and 6,862 probes (1,246 genes) were designed for UCYN-A1 and UCYN-A2, respectively. These probes were replicated 4 times in the 8 × 60K array slides and 13 times in the 4 × 180K array slide, which allowed internal evaluation of signals. The sequences of all oligonucleotide probes were tested *in silico* for possible cross-hybridization as described below. The probe sequences were used as queries in the BLASTN against the following available nt databases in June 2014: Marine microbes, Microbial Eukaryote Transcription, and Non-redundant Nucleotides in the Community Cyberinfrastructure for Advanced Microbial Ecology Research and Analysis (CAMERA; https://www.imicrobe.us [55]). Agilent technology allows 5% nt mismatch in the whole probe region; thus, sequences with a range of 95% to 100% nt identity to the target probe are detected. Therefore, all probes with BLASTN hits with ≥95% over 100% of the nt length were deleted. Next, the probe sequences that passed the cross-hybridization filter were clustered using CD-HIT-EST (56, 57) at 95% nt similarity to select unique probes for UCYN-A1 and unique probes for UCYN-A2. Finally, to select probes specific for each strain, the probes with ≥95% nt identity to the genes in the other strain were deleted. However, a few probes that showed cross-hybridization between the two strains for highly conserved genes (such as the *nifH* nitrogenase gene) were retained. In summary, 6,120 probes for 1,194 genes of UCYN-A1 and 6,324 probes for 1,244 genes of UCYN-A2 were chosen.

In addition, standard control probes (IS-62976-8-V2_60Kby8_GX_EQC_201000210 with ERCC control probes added) were included randomly as part of the Agilent Technology array to feature locations on the microarray slide. The final design of the microarray was synthesized on two platforms: ca. 62,976 experimental probes and 1,319 control probes on the 8 × 60K array slide and ca. 180,880 experimental probes and 4,854 control probes on the 4 × 180K array slide. The probe sequences are available at NCBI Gene Expression Omnibus (GEO) under accession number GSE100124.

### Design of the T. erythraeum IMS101 array.

A custom oligonucleotide array for T. erythraeum was designed using a Roche NimbleGen platform (NimbleGen design identifier [ID]: 080610_Trich_erth_UCSC_TS_expr) according to the complete genome assembly of T. erythraeum IMS101 (NC_008312). The genome sequence is publicly available via on-line gateways, including GenBank (https://www.ncbi.nlm.nih.gov/nuccore/NC_008312), IMG (https://img.jgi.doe.gov/cgi-bin/m/main.cgi?section=TaxonDetail&page=taxonDetail&taxon_oid=637000329), and the genome browser at the University of California, Santa Cruz (UCSC) (http://microbes.ucsc.edu/cgi-bin/hgGateway?db=tricEryt_IMS101). Up to six 60-nt-long tiling probes were designed to target each of the 4,788 genes in the genome, resulting in a total of 28,235 probes. The probes were duplicated on the array to allow internal evaluation of hybridization signals. Moreover, 60-nt oligonucleotide tiling probes were also designed to target the intergenic regions that were >60 bp in length at 150-bp intervals, leading to a total of 11,175 probes targeting 3,877 intergenic regions (average, 2.9 probes per intergenic region); however, hybridization data for intergenic probes are not presented here. All the probes were rank ordered and selected based on the following criteria: (i) a minimum annealing temperature of 68°C; (ii) no cross-contamination among the probes for different genes and for different intergenic regions. In addition to the experimental probes, standard control probes were also included on the microarray for quality assessment of the sample preparation, the hybridization process, and the intensity measurements (see Fig. S3 in the supplemental material). The final microarray slides were printed in 4-plex (4 × 72K), format with 67,645 experimental probe features and 7,454 control probe features on one array. The full microarray platform descriptions and data for T. erythraeum are available at NCBI GEO under accession number GSE99896. Microarray hybridization signals were quantified using a GenePix 4000B scanner (Molecular Devices, Sunnyvale, CA, USA) at the Roche NimbleGen facility.

10.1128/mBio.02495-18.4 FIG S3 Box plot showing that quantile normalization of gene hybridization signals for the T. erythraeum IMS101 arrays resulted in similar signal distribution profiles. Raw hybridization data (A) and normalized data (B) are shown for a total of 28 samples. The different colors correspond to different samples. All samples for T. erythraeum IMS101 hybridization passed the quality control testing. Download FIG S3, PDF file, 1.4 MB. Copyright © 2019 Muñoz-Marin et al. 2019 Muñoz-Marin et al. This content is distributed under the terms of the Creative Commons Attribution 4.0 International license.

### Microarray data analysis.

All data analyses were performed with R (www.R-project.org) and the Bioconductor Project (58), specifically, using the Biobase (59), Linear Models for Microarray LIMMA (60), arrayQualityMetrics (61), affyPLM (62, 63), and genefilter packages.

**(i) UCYN-A microarray.** Transcription values for each gene were obtained using median polish summarization, and values were normalized using quantile normalization (62, 63) (see Fig. S2). The transcription values for UCYN-A at L6, L9, D3, and 2L12 represent the mean transcription values for the two replicates (L6-1, L6-2, L9-1, L9-2, D3-1, D3-2, 2L12-1, and 2L12-2). Raw and normalized microarray data for UCYN-A were submitted to NCBI GEO under accession number GSE100124. To determine if transcription of a gene was detected, the signal-to-noise ratio (SNR) of each chip was calculated as SNR = (*S_i_* – BG)/BG, where *S_i_* is the hybridization signal for the gene and BG is the chip background signal determined as the average of the lowest 5% of all signals. Transcription was considered to have been detected if the SNR of a transcript was ≥5 (as described previously by Shilova et al. [53]). Transcription values were centered and scaled across genes and samples, and a distance matrix was calculated using Pearson’s correlation coefficient. The distance matrix was then used in hierarchical clustering by a complete agglomeration method to identify clusters of genes with similar patterns of transcription during the diel transcription.

10.1128/mBio.02495-18.3 FIG S2 Box plot showing that normalization of gene hybridization signals for the three UCYN-A arrays resulted in similar signal distribution profiles. All samples used for UCYN-A hybridization passed the quality control testing. Raw hybridization probe data (A, B, and C) and quantile-normalized data (D, E, and F**)** are shown for a total of 12 samples hybridized to three different slides. The distribution of hybridization signals is shown for 10,000 randomly sampled probes (including experimental and control probes) for raw data and for 14,000 probes (experimental) for normalized data. *Y*-axis data represent the hybridization signal shown as a log_2_ exponent. Download FIG S2, PDF file, 0.9 MB. Copyright © 2019 Muñoz-Marin et al. 2019 Muñoz-Marin et al. This content is distributed under the terms of the Creative Commons Attribution 4.0 International license.

**(ii) T. erythraeum microarray.** The raw microarray data for T. erythraeum were subjected to robust multichip average (RMA) analysis (64) and quantile normalization (62, 63) (see Fig. S3 in the supplemental material). Transcription values for each gene were obtained using median polish summarization (53). The final transcription value for each sample represented the mean of data from up to 12 technical replicates (blocks 1 and 2, with up to six replicate probes in each block in the T. erythraeum microarray design). A gene was selected for further analysis if it had a log_2_ transcription value above 64 in at least 25% of samples and an interquartile range across all samples on the log_2_ scale of at least 0.5. This filtering resulted in 4,128 genes, which were used in further analysis.

**(iii) Comparison of diel transcription patterns for all cyanobacteria.** Transcription data for *Prochlorococcus* sp. MED4, *Cyanothece* sp. ATCC 51142, and Crocosphaera watsonii WH 8501 were collected from previously published data (16 – 18). *Cyanothece* sp. ATCC 51142 and C. watsonii WH 8501 microarray data were downloaded from ArrayExpress (http://www.ebi.ac.uk/aerep/) using accession no. E-TABM-386 and E-TABM-737, respectively. The genes with periodic transcriptional patterns for all cyanobacteria studied here (*Prochlorococcus* sp. MED4, *Cyanothece* sp. ATCC 51142, C. watsonii WH 8501, T. erythraeum, and UCYN-A) were identified using the R package “cycle” based on Fourier analysis, and the genes with a false-discovery rate (FDR) of <0.25 were selected for further comparison (65) (Table S2). To compare the diel transcription patterns among the cyanobacteria, gene transcription values for each cyanobacterium were selected for over 36 h. Eight points were selected for UCYN-A (L6, L9, D3, D6, 2D12, 2L3, 2L9, and 2L12), 9 points for T. erythraeum (D12, L3, L6, L9, L12, D3, D6, D9, and 2D12), 6 points for *Cyanothece* sp. ATCC 51142 (L2, L6, L10, D2, D6, and D10), 8 points for C. watsonii WH 8501 (D11, L1, L6, L11, D1, D6, 2D11, and 2L1) and 19 points for *Prochlorococcus* sp. MED4 (D12 to 2L12 [every 2 h]). “L” and “D” stand for the light period and the dark period, respectively, “2L” and “2D” for the second light-dark cycle, and the final number for the hour corresponding to entry into the light or dark period. Because the studies had a few dissimilar sampling times, the missing values were interpolated using the Stineman algorithm implemented in the *imputeTS* package (66). A network was constructed based on the Pearson correlation and the ‘make_network’ function in phyloseq (67). The maximum distance between connecting nodes was selected as 0.5 unless otherwise noted in the figure legends.

**Accession number(s).** Microarray data have been deposited at the NCBI Gene Expression Omnibus (GEO) under accession numbers GSE100124 and GSE99896.
