# Supplementary figures and images for: The Transcriptional Cycle Is Suited to Daytime N2 Fixation in the Unicellular Cyanobacterium “Candidatus Atelocyanobacterium thalassa” (UCYN-A)
Source: mBio. 2019 Jan 2;10(1):e02495-18. doi: 10.1128/mBio.02495-18 (PMC6315102; doi:10.1128/mBio.02495-18)

**
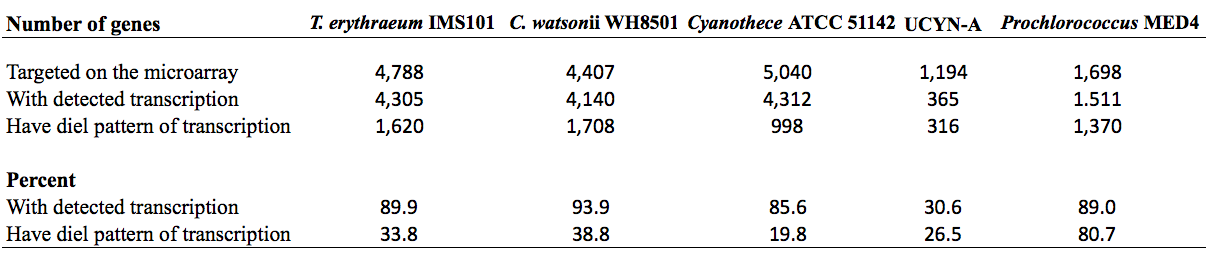
**

Supplement: TABLE S1 [file mbo004184246st1.docx]

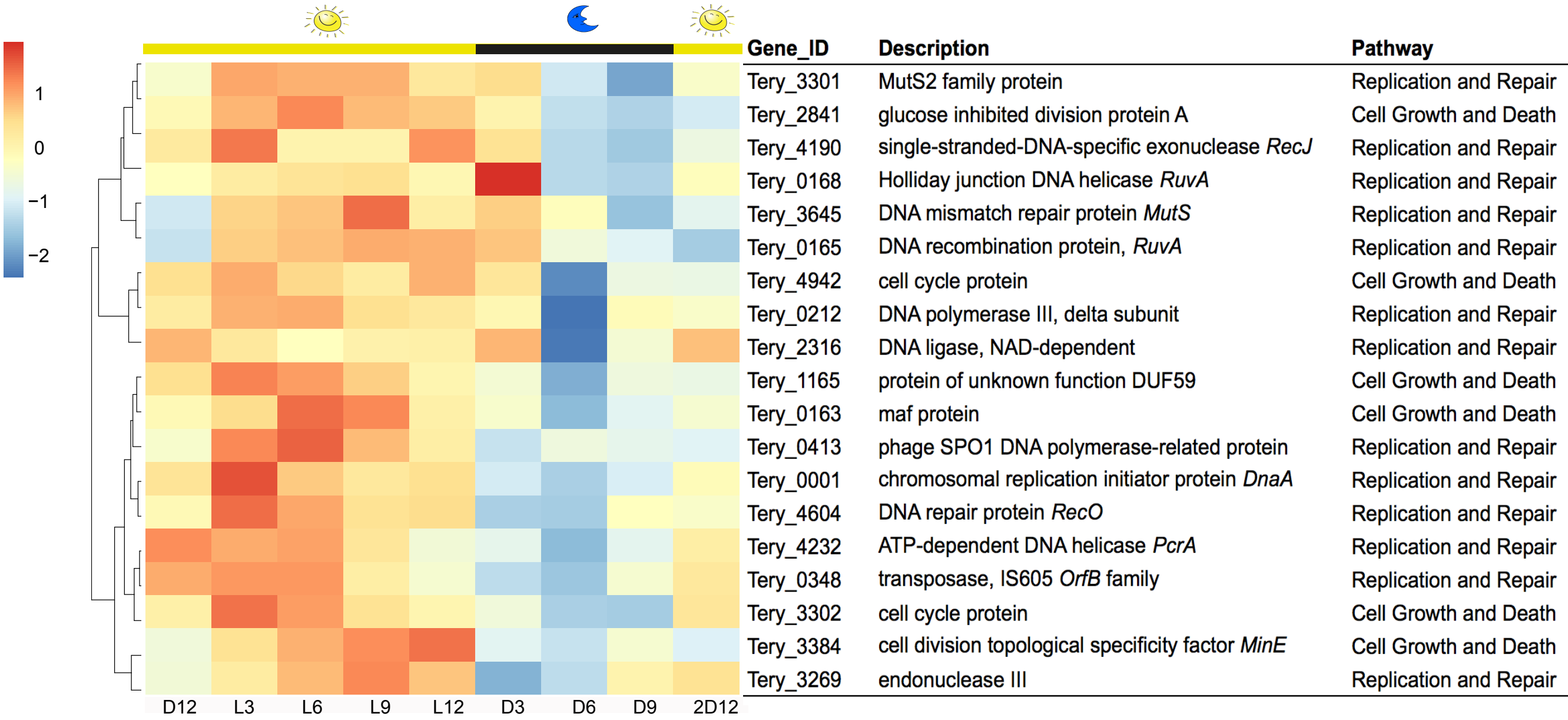

Supplement: FIG S1 [file mbo004184246sf1.pdf]

**
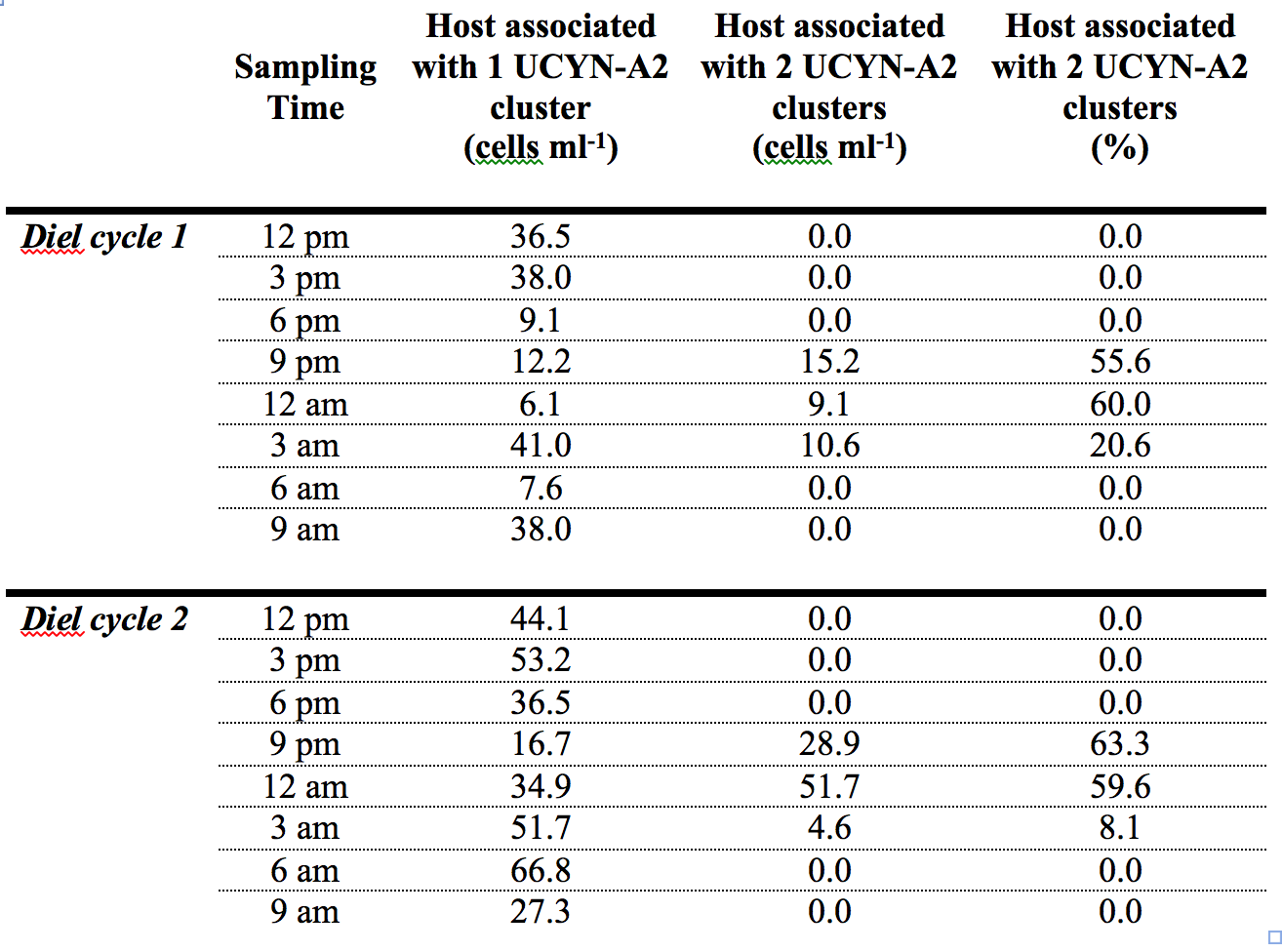
**

Supplement: TABLE S6 [file mbo004184246st6.docx]

**A.**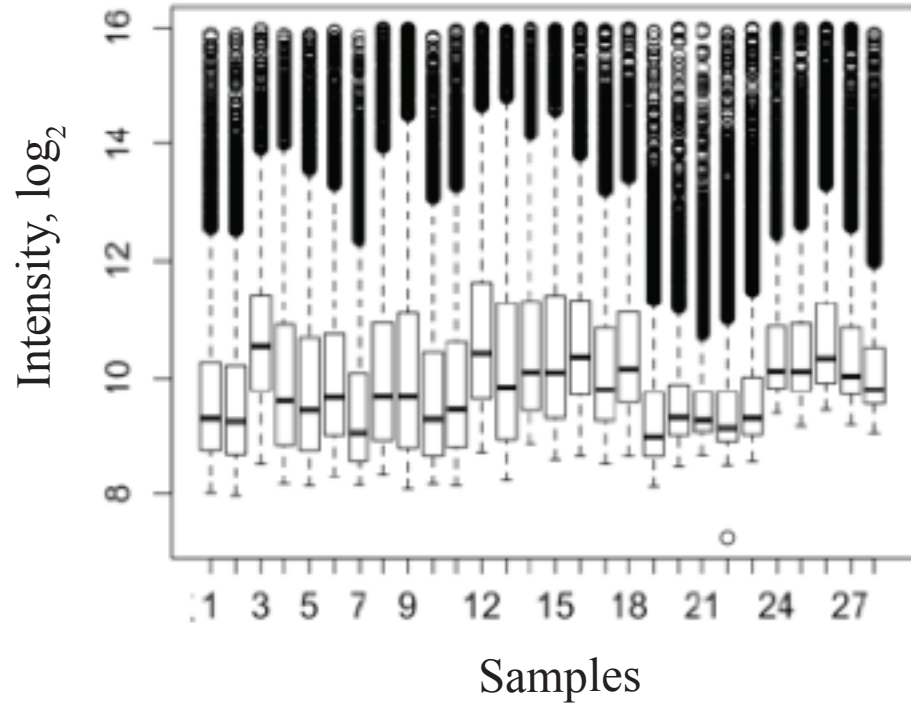**B.**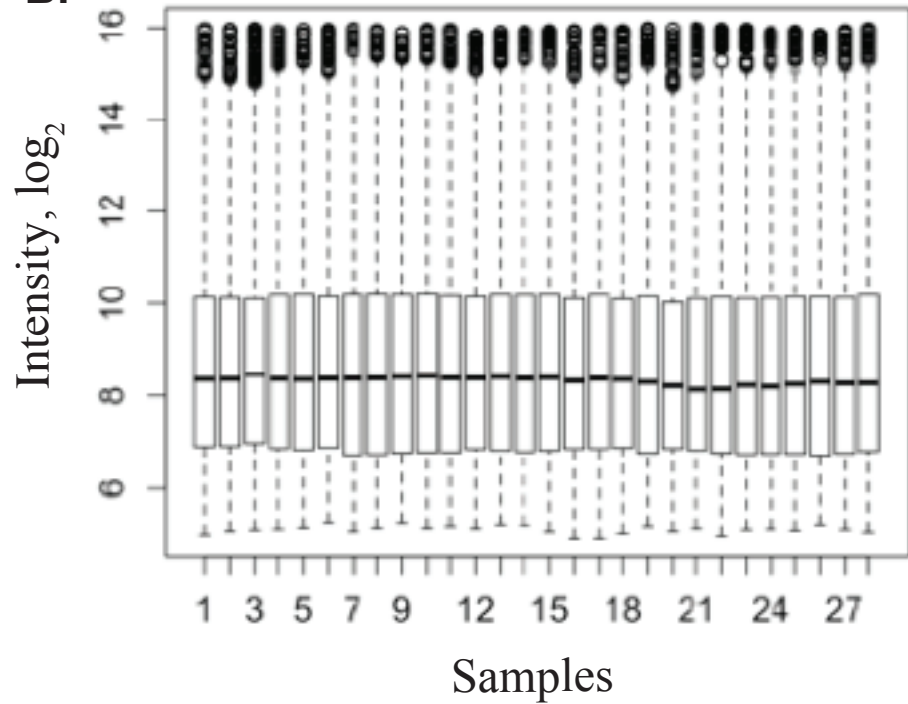

Supplement: FIG S3 [file mbo004184246sf3.pdf]

A.

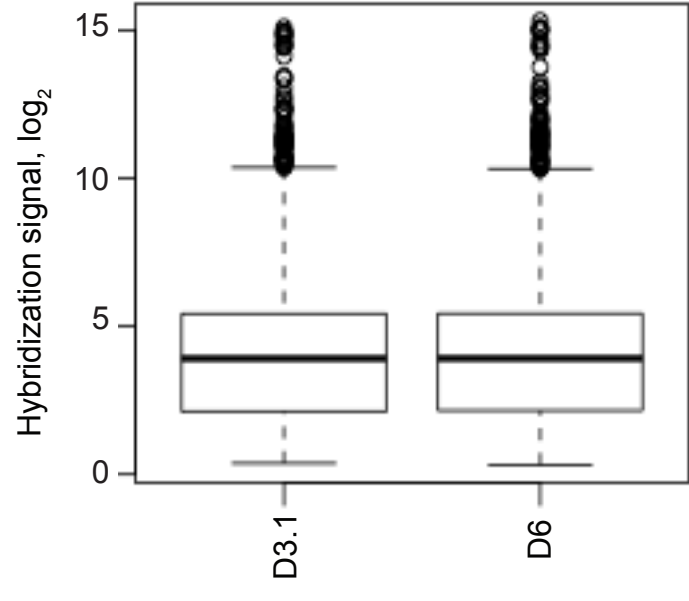

B.

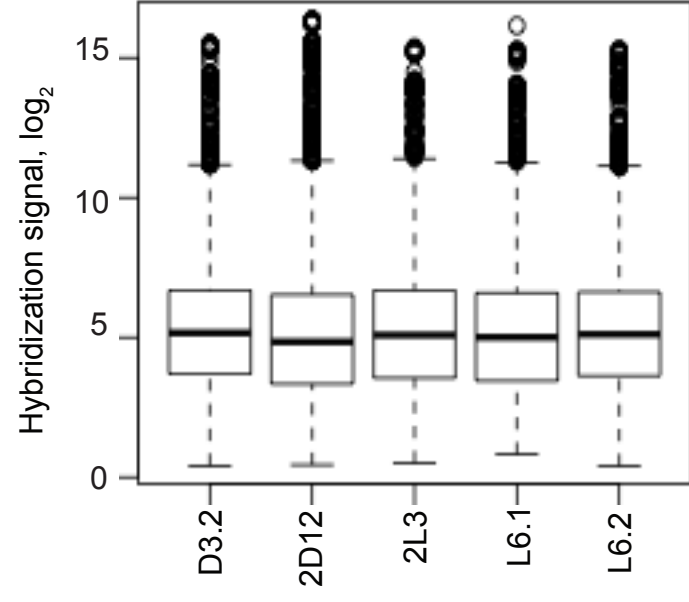

C.

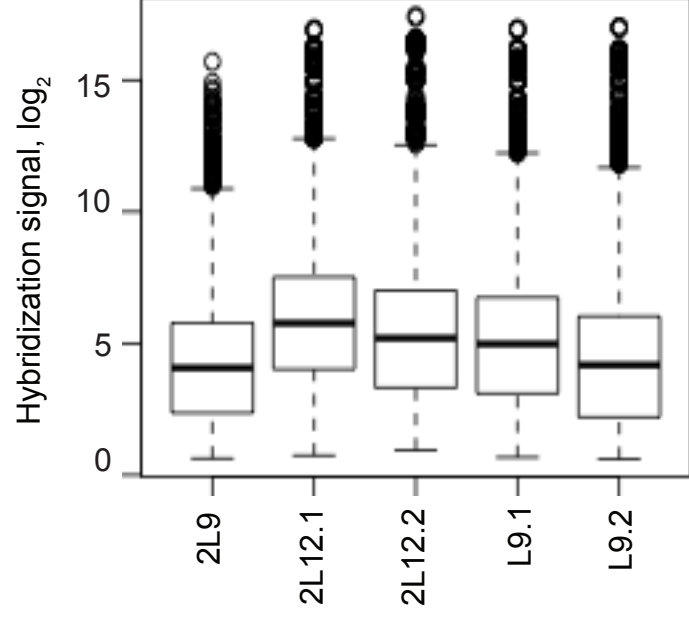

D.

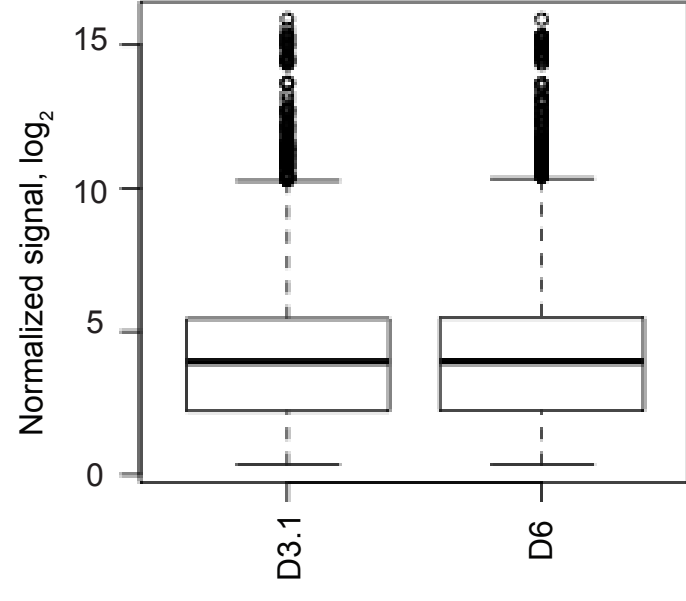

E.

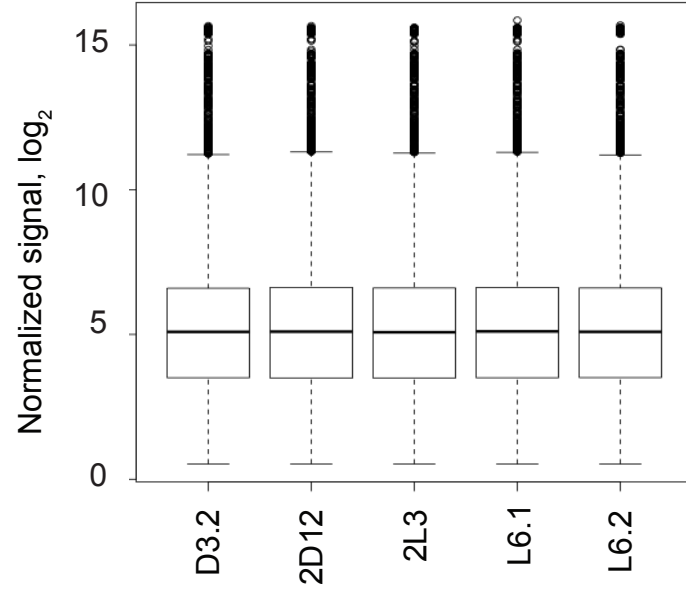

F.

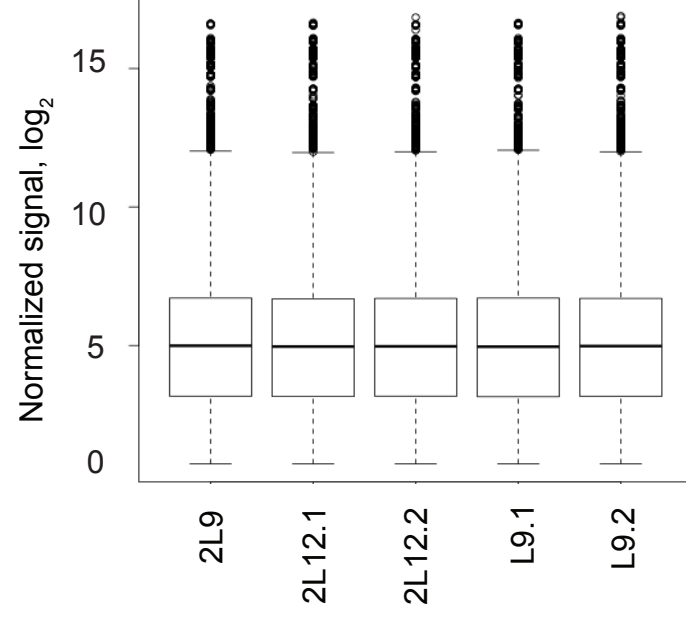

Supplement: FIG S2 [file mbo004184246sf2.pdf]
