# Supplementary material for: The Transcriptional Cycle Is Suited to Daytime N2 Fixation in the Unicellular Cyanobacterium “Candidatus Atelocyanobacterium thalassa” (UCYN-A)
Source: mBio. 2019 Jan 2;10(1):e02495-18. doi: 10.1128/mBio.02495-18 (PMC6315102; doi:10.1128/mBio.02495-18)
Supplement: TEXT S1 [file mbo004184246s1.docx]

**Supplementary Information**

Extended description of Material and Methods described in the main text.

**Double CARD-FISH assay**

Filters were embedded in 0.1% Ultrapure Agarose (Invitrogen, ThermoFisher, Waltham, MA, USA) to minimize cell loss and incubated at 35ºC for 10-30 min until the agarose was completely dry. The filters were treated with 100 mg lysozyme (37°C, 1 h) and 60 U/mL achromopeptidase (37°C, 30 min) to permeabilize cell walls. The treated filters were then cut into 8 equal slices.

We used competitor probes for specific detection of different species in environmental samples to avoid cross-hybridization with other closely related species (1-4). The competitor probes are unlabeled oligonucleotides which are fully complementary to the mismatch-containing non-target sequence. Moreover, we used helper probes to increase the access of the probe to the 16S rRNA target regions, by binding near the probe target sequence (5, 6). All of the probes, competitors and helpers used in this work are compiled in Table S7.

We used either the probe UPRYM69, which is specific for the UCYN-A1 host, or the UBRADO69 probe, specific for the UCYN-A2 host (Table S7), in the first round of each hybridization. For the first hybridization, filter pieces (1/8 of the filter) were covered with 12 µL hybridization buffer (40% deionized formamide, 0.9 mol L^-1^ NaCl, 20 mmol L^-1^ Tris-HCl pH 8, 0.01% sodium dodecyl sulfate (SDS), 20 mg mL^−1^ blocking reagent (Roche Diagnostic Boehringer, Basel, Switzerland), and 5 ng μL^–1^ of the desired probe (UPRYM69 or UBRADO69), competitor probe and the helper probes (Helper A-PRYM or Helper B-PRYM) and incubated at 46°C overnight. The filters were then incubated at 48°C with the washing buffer (56 mmol L^-1^ NaCl, 5 mmol L^-1^ EDTA, 0.01% SDS, 20 mmol L^-1^ Tris-HCl pH 8) twice, for 10 min in each wash step. After the washing steps, the filters were equilibrated in 1% phosphate-buffered saline (PBS) buffer for 15 min at room temperature.

Tyramide signal amplification (TSA) was done for 10 min in the dark with TSA™Alexa Fluor ® 488 Tyramide following the instructions of the manufacturer (Molecular probes, Eugene, OR, USA). Following amplification, samples were equilibrated with 1% PBS solution for 20 min, rinsed twice with MilliQ water and air-dried. Before the second CARD-FISH hybridization, probe peroxidases were inactivated with 0.01 mol L^-1^ HCl for 10 min at room temperature in the dark, then filters were rinsed twice with MilliQ water and air dried.

For the second hybridization, we used the probe UCYNA1-732 for UCYN-A1 or UCYN-A2-732 for UCYN-A2 with the competitor probe for UCYN-A1 and UCYN-A2 respectively, and the helper probes (HelperA-732 or HelperB-732) at 0.16 ng μL^–1^ each (6) (Table S7). Filters were embedded in the hybridization buffer (50% formamide, 0.9 mol L^-1^ NaCl, 20 mmol L^-1^ Tris-HCl pH 8, 0.01% SDS, 10 mg mL^−1^ blocking reagent and 100 mg mL^− 1^ dextran sulfate) with the probe and helpers and incubated at 35°C for 3 hours.

After the hybridization, the filters were rinsed in washing buffer (9 mmol L^-1^ NaCl, 5 mmol L^-1^ EDTA, 0.01% SDS, 20 mmol L^-1^ Tris-HCl pH 8) at 37°C, and the TSA reaction was done using TSA™ Plus Cyanine 3 System (Perkin Elmer, Inc, Waltham, MA, USA) for 10 min at room temperature in the dark following the manufacturer’s instructions.

Filters were stained with 5 μg mL^–1^ DAPI (4′, 6- diamidino-2-phenylindole), mounted in antifading reagent (77% glycerol, 15% VECTASHIELD and 8% 20 Å~ PBS), and micrographs were obtained using a Leica SP5 Confocal Microscope (Leica Microsystems, Mannheim, Germany) at the University of California, Santa Cruz Life Sciences Microscopy Center. Filters were observed under ultraviolet (for DAPI), blue light (for host stained with Alexa 488) and green light (for UCYN-A stained with Cy3) excitations. Microscopic evaluation and counting was performed with the Carl Zeiss Axioplan-2 Imaging Fluorescent Microscope (Zeiss, Berlin, Germany) in 3 transects (8.0 x 0.1 mm_2_ each) across the filter piece. Cell dimensions were estimated using AxioVision 4.8 and Image J software^40^.

**Design of the UCYN-A array**

The oligonucleotide expression array of UCYN-A was designed using UCYN-A1 and UCYN-A2 genes using eArray web-based tool (Agilent Technology Inc.; https://earray.chem.agilent.com/earray/) similar to the array design described in Shilova et al. (7). The gene sequences were obtained from the National Center of Biotechnology Information (NCBI, <http://www.ncbi.nlm.nih.gov>). Briefly, six probes of 60 nucleotides (nt) length were designed for each gene, and a total of 6618 probes (1199 genes) and 6862 probes (1246 genes) were designed for UCYN-A1 and UCYN-A2, respectively. These probes were replicated (4 times in the 8x60K array slides and 13 times in the 4x180K array slide) which allowed internal evaluation of signals. The sequences of all oligonucleotide probes were tested *in silico* for possible cross-hybridization as described below. The probe sequences were used as queries in the BLASTN against the available nt databases in June 2014: Marine microbes, Microbial Eukaryote Transcription and Non-redundant Nucleotides in the Community Cyberinfrastructure for Advanced Microbial Ecology Research and Analysis (CAMERA, <http://camera.calit2.net/>, (8)). Agilent technology allows 5% nt mismatch in the whole probe region, thus sequences with a range of 95–100% nt identity to the target probe are detected. Therefore, all probes with BLASTN hits with ≥95% over 100% nt length were deleted. Next, probe sequences that passed the cross-hybridization filter, were clustered using CD-HIT-EST (9, 10) at 95% nt similarity to select unique probes for UCYN-A1 and unique probes for UCYN-A2. Finally, to select probes specific for each strain, the probes with ≥95% nt identity to the genes in the other strain were deleted. However, a few probes that showed cross-hybridization between both strains for highly conserved genes (such as the nitrogenase gene, *nifH*) were retained. In summary, 6120 probes for 1194 genes of UCYN-A1 and 6324 probes for 1244 genes of UCYN-A2 were chosen.

In addition, standard control probes as part of the Agilent Technology Array (IS-62976-8-V2_60Kby8_GX_EQC_201000210 with ERCC control probes added) were included randomly to feature locations on the microarray slide. The final design of the microarray was synthesized on two platforms: ca. 62976 experimental and 1319 control probes on the 8x60K array slide and ca. 180880 experimental and 4854 control probes on the 4x180K array slide. The probe sequences are available at NCBI Gene Expression Omnibus (GEO) under accession number GSE100124.

**Design of the T. erythraeum IMS101 array**

A custom oligonucleotide array for *T. erythraeum*was designed using the Roche NimbleGen platform: (NimbleGen design ID: 080610_Trich_erth_UCSC_TS_expr) according to the complete genome assembly of *T. erythraeum* IMS101 (NC_008312). The genome sequence is publically available via gateways including GenBank (<https://www.ncbi.nlm.nih.gov/nuccore/NC_0083120>), IMG (<http://img.jgi.doe.gov:80/cgibin/pub/main.cgi?section=TaxonDetail&page=taxonDetail&taxon_oid=637000329>), and UCSC genome browser (<http://microbes.ucsc.edu/cgi-bin/hgGateway?db=tricEryt_IMS101>). Up to six 60-nt long tiling probes were designed to target each of the 4788 unique genes in the genome, resulting in a total of 28235 probes. The probes were duplicated on the array to allow internal evaluation of hybridization signals. Moreover, tiling 60 nt oligonucleotide probes were also designed to target the intergenic regions >60 bp in length at a 150 bp interval, leading to a total of 11175 probes targeting 3877 intergenic regions (average 2.9 probes per intergenic region), however hybridization data for intergenic probes are not presented here. All the probes were rank ordered and selected based on the following criteria: 1) they must have a minimum annealing temperature of 68ºC; 2) there is no cross contamination among the probes for different genes and for different intergenic regions. In addition to the experimental probes, standard control probes were also included on the microarray for quality assessment of the sample preparation, the hybridization process and the intensity measurements. The final microarray slides were printed in 4-plex (4x72K) format with 67645 experimental probe features and 7454 control probe features on one array. The full microarray platform descriptions and data for *T. erythraeum* are available at NCBI GEO under accession number GSE99896. Microarray hybridization signals were quantified using a GenePix 4000B Scanner (Molecular Devices, Sunnyvale, CA, USA) at the Roche NimbleGen facility.

**Microarray data analysis**

All data analyses were performed with R (www. R-project.org) and the Bioconductor Project (11), specifically using the Biobase (12), Linear Models for Microarray LIMMA (13), arrayQualityMetrics (14), affyPLM (15, 16), and genefilter packages.

*UCYN-A microarray*

Transcription values for each gene were obtained using median polish summarization, and values were normalized using quantile normalization (15, 16) (Figure S2). The transcription values for UCYN-A at L6, L9, D3 and 2L12 are the mean transcription of the two replicates (L6-1, L6-2, L9-1, L9-2, D3-1, D3-2, 2L12-1 and 2L12-2). Raw and normalized microarray data for UCYN-A were submitted to NCBI GEO under accession number GSE100124. To determine if transcription of a gene was detected, the signal-to-noise ratio (SNR) of each chip was calculated as: SNR = (S_i_–BG)/BG; where S_i_ is the hybridization signal for the gene and BG is the chip background signal determined as average of the lowest 5% of all signals. Transcription was considered detected if SNR of a transcript was ≥5 (as in (Shilova et al. 2014). Transcription values were centered and scaled across genes and samples, and a distance matrix was calculated using Pearson’s correlation coefficient. The distance matrix was then used in hierarchical clustering by a complete agglomeration method to identify clusters of genes with similar patterns of transcription during the diel transcription.

*T. erythraeum microarray*

The raw microarray data for *T. erythraeum* were subjected to robust multichip average (RMA) analysis (17) and quantile normalization (15, 16) (Figure S3). Transcription values for each gene were obtained using median polish summarization (7). Final transcription value for each sample was a mean of up to twelve technical replicates (Blocks 1 and 2 with up to six replicate probes in each block in the *T. erythraeum* microarray design). The genes with detected transcription were determined as described above for the UCYN-A microarray.

*Comparison of diel transcription patterns for all cyanobacteria*

Transcription data for *Prochlorococcus* sp. MED4, *Cyanothece* sp. ATCC 51142 and *Crocosphaera watsonii* WH 8501 were collected from previous published data (18-20). *Cyanothece* sp. ATCC 51142 and *C. watsonii* WH 8501 microarray data were downloaded from ArrayExpress (<http://www.ebi.ac.uk/aerep/>) using accession no. E-TABM-386 and E-TABM-737, respectively. The genes with periodic transcriptional patterns for all studied cyanobacteria (*Prochlorococcus* sp*.* MED4, *Cyanothece* sp. ATCC 51142, *C. watsonii* WH 8501, *T. erythraeum* and UCYN-A) were identified using the R package “cycle” based on Fourier analysis. The False Discovery Rate (FDR) was chosen based on the distribution of the Fourier scores (21). In the case of *C. watsonii* WH 8501 and *Cyanothece* sp. ATCC 5114 the genes with FDR≤0.30 were selected for further comparison, while all the rest of organisms have genes with FDR FDR≤0.25 (Table S2). To compare the diel transcription patterns among the cyanobacteria, gene transcription values for each cyanobacterium were selected for over 36 hours. Eight points were selected for UCYN-A (L6, L9, D3, D6, 2D12, 2L3, 2L9, 2L12), 9 points for *T. erythraeum* (D12, L3, L6, L9, L12, D3, D6, D9, 2D12), 6 points for *Cyanothece* sp. ATCC 51142 (L2, L6, L10, D2, D6, D10), 8 points for *C. watsonii* WH 8501 (D11, L1, L6, L11, D1, D6, 2D11, 2L1) and 19 points for *Prochlorococcus* sp. MED4 (D12 - 2L12 every 2 hours). L and D stand for light and dark period, respectively, 2L and 2D the second light-dark cycle, and the number the corresponding hours entering light or dark period. Because the studies had a few dissimilar sampling times, the missing values were interpolated using the Stineman algorithm implemented in the *imputeTS* package (22). A network was constructed based on the Pearson correlation and using ‘make_network’ function in phyloseq (23). The maximum distance between connecting nodes was selected as 0.5 unless otherwise noted in figure legends.

**References for supplemental information**

1. Manz W, Amann R, Ludwig W, Wagner M, Schleifer K. 1992. Phylogenetic oligodeoxynucleotide probes for the major subclasses of *Proteobacteria* problems and solutions. Syst Appl Microbiol 15:593-600.

2. Lin X, Wakeham S, Putnam I, Astor Y, Scranton M, Chistoserdov A, Taylor G. 2006. Comparison of vertical distributions of prokaryotic assemblages in the anoxic Cariaco Basin and Black Sea by use of fluorescence in situ hybridization. Appl Environ Microbiol 72:2679-2690.

3. Kubota K, Ohashi A, Imachi H, Harada H. 2006. Visualization of *mcr* mRNA in a methanogen by fluorescence in situ hybridization with an oligonucleotide probe and two-pass tyramide signal amplification (two-pass TSA-FISH). J Microbiol Methods 66:521-528.

4. Ishii K, Muβmann M, MacGregor B, Amann RI. 2004. An improved fluorescence in situ hybridization protocol for the identification of bacteria and archae in marine sediments. FEMS Microbiol Ecol 50:203-213.

5. Krupke A, Musat N, LaRoche J, Mohr W, Fuchs BM, Amann RI, Kuypers MM, Foster R. 2013. In situ identification and N_2_ and C fixation rates of uncultivated cyanobacteria populations. Syst Appl Microbiol 36:259-271.

6. Cornejo-Castillo FM, Cabello AM, Salazar G, Sánchez-Baracaldo P, Lima-Mendez G, Hingamp P, Alberti A, Sunagawa S, Bork P, De Vargas C, Raes J, Bowler C, Wincker P, Zehr JP, Gasol JM, Massana R, Acinas SG. 2016. Cyanobacterial symbionts diverged in the late Cretaceous towards lineage-specific nitrogen fixation factories in single-celled phytoplankton. Nature Communications 7:1-9.

7. Shilova IN, Robidart JC, Tripp HJ, Turk-Kubo K, Wawrik B, Post AF, Thompson AW, Ward B, Hollibaugh JT, Millard A. 2014. A microarray for assessing transcription from pelagic marine microbial taxa. The ISME Journal 8:1476-1491.

8. Sun S, Chen J, Li W, Altinatas I, Lin A, Peltier S, Stocks K, Allen EE, Ellisman M, Grethe J, Wooley J. 2011. Community cyberinfrastructure for Advanced Microbial Ecology Research and Analysis: the CAMERA resource Nucl Acids Res 39:D546-D551.

9. Huang Y, Niu B, Gao Y, Fu L, Li W. 2010. CD-HIT Suite: a web server for clustering and comparing biological sequences. Bioinformatics 26:680-682.

10. Li W, Godzik A. 2006. Cd-hit: a fast program for clustering and comparing large sets of protein or nucleotide sequences. Bioinformatics 22:1658 - 1659.

11. Gentleman R, Carey V, Bates D, Bolstad B, Dettling M, Dudoit S, Ellis B, Gautier L, Ge Y, Gentry J, Hornik K, Hothorn T, Huber W, Iacus S, Irizarry R, Leisch F, Li C, Maechler M, Rossini A, Sawitzki G, Smith C, Smyth G, Tierney L, Yang J, Zhang J. 2004. Bioconductor: open software development for computational biology and bioinformatics. Genome Biol 5:R80.

12. Huber W, Carey JV, Gentleman R, Anders S, Carlson M, Carvalho SB, Bravo CH, Davis S, Gatto L, Girke T, Gottardo R, Hahne F, Hansen DK, Izarry AR, Lawrence M, Love IM, Waldron L, Morgan. 2015. Orchestrating high-throughput genomic analysis with Bioconductor. Nat Meth 12:115121.

13. Smyth GK. 2005. Limma: Linear models for microarray data., p 397-420. *In* Gentleman R, Carey V, Dudoit S, Irizarry R, Huber W (ed), Bioinformatics and Computational Biology Solutions using R and Bioconductor. Springer, New York.

14. Kauffmann A, Gentleman R, Huber W. 2009. arrayQualityMetrics-a bioconductor package for quality assessment of microarray data. Bioinformatics 25:415-416.

15. Bolstad BM, Collin F, Brettschneider J, Simpson K, Cope L, Irizarry RA, Speed TP. 2005. Quality Assessment of Affymetrix GeneChip Data, p 33-47. *In* Springer NY (ed), Bioinformatics and Computational Biology Solutions using R and Bioconductor.

16. Bolstad B. 2004. Low level analysis of high-density oligonucleotide Array Data: Background, Normalization and SummarizationUniversity of California, Berkeley.

17. Irizarry R, Hobbs B, Collin F, Beazer-Barclay Y, Antonellis K, Scherf U, Speed T. 2003. Exploration, normalization, and summaries of high density oligonucleotide array probe level data. Biostatistics 4:249.

18. Shi T, Ilikchyan I, Rabouille S, Zehr JP. 2010. Genome-wide analysis of diel gene expression in the unicellular N_2_-fixing cyanobacteria *Crocosphaera watsonii* WH 8501. ISME J 4:621-632.

19. Zinser ER, Lindell D, Johnson ZI, Futschik ME, Steglich C, Coleman ML, Wright MA, Rector T, Steen R, McNulty NP, Thompson LR, Chisholm SW. 2009. Choreography of the transcriptome, photophysiology, and cell cycle of a minimal photoautotroph, *Prochlorococcus*. PLoS ONE 4:e5135.

20. Toepel J, Welsh E, Summerfield T, Pakrasi H, Sherman L. 2008. Differential transcriptional analysis of the cyanobacterium *Cyanothece* sp. strain ATCC 51142 during light-dark and continuous-light growth. J Bacteriol 190:3904-3913.

21. Futschik ME, Herzel H. 2008. Are we overestimating the number of cell-cycling genes? The impact of background models on time series analysis. Bioinformatics 24:1063-1069.

22. Moritz S, Sardá A, Bartz-Beielstein T, Zaefferer M, Stork J. 2015. Comparison of different methods for univariate time series imputation in R. arXiv:151003924

23. McMurdie P, Holmes S. 2013. Phyloseq: An R package for reproducible interactive analysis and graphics of microbiome census data. PLoS ONE 8:e61217.
