# Supplementary material for: The Transcriptional Cycle Is Suited to Daytime N2 Fixation in the Unicellular Cyanobacterium “Candidatus Atelocyanobacterium thalassa” (UCYN-A)
Source: mBio. 2019 Jan 2;10(1):e02495-18. doi: 10.1128/mBio.02495-18 (PMC6315102; doi:10.1128/mBio.02495-18)
Supplement: TABLE S7 [file mbo004184246st7.docx]

| Probe Name | Target | Sequence (5’ to 3’) | Reference |
| --- | --- | --- | --- |
| UPRYM69 | Host-A1 | CACAT**A**GGAACATCCTCC | Cornejo-Castillo et al.(6) |
| UPRYM69 competitor | Host-A2 used as  Host-A1 competitor | CACAT**T**GGAACATCCTCC | Cornejo-Castillo et al.(6) |
| UBRADO69 | Host-A2 | CACAT**T**GGAACATCCTCC | Cornejo-Castillo et al.(6) |
| UBRADO69 competitor | Host-A1 used as  Host-A2 competitor | CACAT**A**GGAACATCCTCC | Cornejo-Castillo et al.(6) |
| Helper A-PRYM | *Haptophyta* | GAAAGGTGCTGAAGGAGT | Cornejo-Castillo et al.(6) |
| Helper B-PRYM | *Haptophyta* | AATCCCTAGTCGGCATGG | Cornejo-Castillo et al.(6) |
| UCYN-A1 732 | UCYN-A1 | GTT**A**CGGTCCAGTAGCAC | Krupke et al.(5) |
| UCYN-A1 competitor | UCYN-A2 used  as UCYN-A1 competitor | GTT**G**CGGTCCAGTAGCAC | Cornejo-Castillo et al.(6) |
| UCYN-A2 732 | UCYN-A2 | GTT**G**CGGTCCAGTAGCAC | Cornejo-Castillo et al.(6) |
| UCYN-A2 competitor | UCYN-A1 used  as UCYN-A2 competitor | GTT**A**CGGTCCAGTAGCAC | Krupke et al.(5) |
| Helper A-732 | UCYN-A | GCCTTCGCCACCGATGTTCTT | Krupke et al.(5) |
| Helper B-732 | UCYN-A | AGCTTTCGTCCCTGAGTGTCA | Krupke et al.(5) |
